# Microglial activation increases cocaine self-administration following adolescent nicotine exposure

**DOI:** 10.1038/s41467-019-14173-3

**Published:** 2020-01-16

**Authors:** K. E. Linker, M. Gad, P. Tawadrous, M. Cano, K. N. Green, M. A. Wood, F. M. Leslie

**Affiliations:** 1 Department of Anatomy and Neurobiology, University of California Irvine, Irvine, CA USA; 2 Department of Neurobiology and Behavior, University of California Irvine, Irvine, CA USA; 3 Department of Pharmacology, University of California Irvine, Irvine, CA USA

**Keywords:** Glial development, Neuroimmunology, Reward

## Abstract

With the rise of e-cigarette use, teen nicotine exposure is becoming more widespread. Findings from clinical and preclinical studies show that the adolescent brain is particularly sensitive to nicotine. Animal studies have demonstrated that adolescent nicotine exposure increases reinforcement for cocaine and other drugs. However, the mechanisms that underlie these behaviors are poorly understood. Here, we report reactive microglia are critical regulators of nicotine-induced increases in adolescent cocaine self-administration. Nicotine has dichotomous, age-dependent effects on microglial morphology and immune transcript profiles. A multistep signaling mechanism involving D2 receptors and CX3CL1 mediates nicotine-induced increases in cocaine self-administration and microglial activation. Moreover, nicotine depletes presynaptic markers in a manner that is microglia-, D2- and CX3CL1-dependent. Taken together, we demonstrate that adolescent microglia are uniquely susceptible to perturbations by nicotine, necessary for nicotine-induced increases in cocaine-seeking, and that D2 receptors and CX3CL1 play a mechanistic role in these phenomena.

## Abstract (web summary)

Adolescents are particularly sensitive to nicotine. Here the authors show that in mice, microglial activation contributes to the enhanced sensitivity to cocaine caused by nicotine exposure in young mice.

## Introduction

Tobacco use typically begins in adolescence, with ~90% of adult smokers starting before the age of 18^1^. Smoking during early adolescence is associated with significantly increased risk of future cocaine abuse^2–4^. While tobacco use has declined in recent decades, teen e-cigarette use has increased at an exponential rate, and the percentage of high-school e-cigarette smokers recently surged to over 30%^5,6^. Teens that smoke e-cigarettes have a higher propensity to initiate other substance use, including cocaine^7^. Several groups have shown that adolescent, but not adult, rodent nicotine exposure causes increases in cocaine-associated behaviors^8–13^. These observations demonstrate that adolescents are uniquely sensitive to nicotine. Therefore, understanding the distinct effects of nicotine use on adolescents is critical for treating and preventing the adverse health consequences that follow.

Adolescence is the final developmental stage before adulthood, with hallmark changes to dopaminergic circuitry and synaptic pruning^14–16^. Regions that receive dopaminergic innervation, such as the nucleus accumbens (NAc), basolateral amygdala (BLA), and prefrontal cortex (PFC), are among the last brain circuits to fully mature in both humans and rodents^16^. Within the NAc and PFC, dopamine D2 receptors are functionally immature^17,18^. The adolescent brain also experiences a pruning of select synapses, with a concurrent strengthening of remaining connections^15,16,19,20^.

Nicotine interferes with adolescent brain maturation, and causes persistent changes in neuronal signaling^15,16^. Adolescent-nicotine exposure modifies cortico-limbic signaling^8,15^, increases D2 receptor activity^9,21^, and restructures synaptic pruning patterns in reward-encoding brain regions^14,16^. Although it is well documented that nicotine has unique effects on the adolescent brain, how these neurobiological changes contribute to increases in adolescent cocaine use remains poorly understood^2,8^.

Microglia are the resident immune cells of the brain and contribute to essential aspects of early brain development^22,23^. These cells are critical for developmental synaptic refinement, and prune synapses in an activity-dependent manner^24–29^. Microglia exclusively express the receptor CX3CR1, which mediates developmental synaptic pruning through the neuronal ligand CX3CL1^30^. However, it is unclear if microglia contribute to adolescent synaptic pruning, especially in response to nicotine exposure. Nearly all drugs of abuse acutely activate microglia^31^, leading to a pro-inflammatory state which then alters neurocircuits associated with reward and dependence^30–32^. Inhibiting this inflammatory state can inhibit the rewarding effects of addictive drugs^29–32^. Nicotine is distinct in that has been shown to suppress reactive microglia in the adult brain^33–35^, but it is unclear if this suppressive effect is also present in adolescent microglia, which are more sensitive to immune insults than adult^29^.

Since the vast majority of smokers initiate during adolescence, it is critical to investigate changes that occur in the adolescent brain after nicotine exposure. Here, we have explored the unique effects of nicotine on adolescent microglia, the importance of microglia in nicotine-induced increases in cocaine reinforcement, and the underlying mechanistic role of neuronal D2 receptors and CX3CL1.

## Results

### Nicotine exposure has age-dependent effects on cocaine self-administration and microglia

**Fig. 1:**
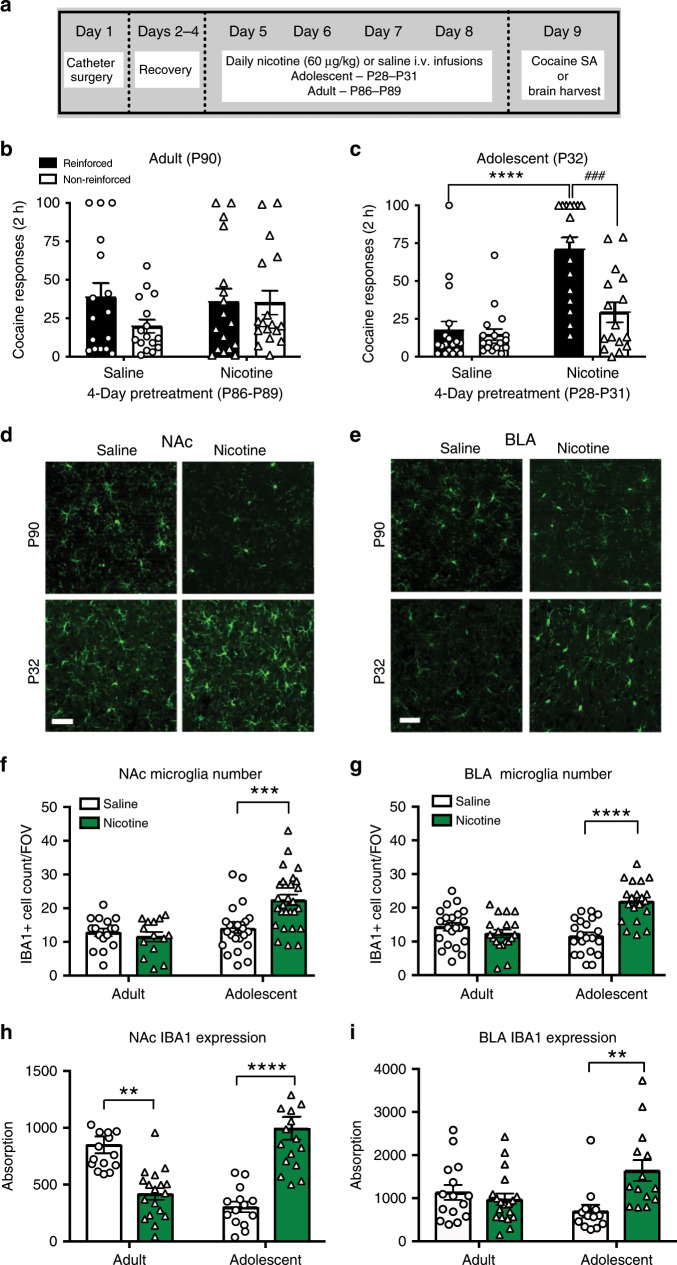
Nicotine increases cocaine responding and microglia in adolescent, but not adult rats. **a** Schematic of nicotine pretreatment paradigm. No significant sex differences in were observed in either adult or adolescent cocaine responding, as such behavioral data are collapsed across sex. **b** Adult nicotine pretreatment did not alter cocaine responding (*F*(1,31) = 0.4876, *p* = 0.4902; *η*^2^ = 0.93%; *n* = 16 saline; 17 nicotine). **c** Adolescent-nicotine exposure increased cocaine responding (*F*(1,32) = 20.75, *p* < 0.0001; *η*^2^ = 26.33%). A significant effect of response (*F*(1,32) = 24.29, *p* < 0.0001; *η*^2^ = 11.09%) and interaction between response and nicotine (*F*(1,32) = 18.48, *p* = 0.0002; *η*^2^ = 8.44%) was observed. Adolescent rats distinguished between reinforced and non-reinforced holes (###*p* = 0.0004). (*n* = 18 saline, 21 nicotine). **d**, **e** Representative images of adult (P90) and adolescent (P32) IBA1 expression in the NAc (**d**) and BLA (**e**). No sex **d**ifferences were observed in adult or adolescent microglial expression or count. **f** Nicotine exposure increased adolescent NAc microglia number (*F*(1,66) = 7.828, *p* = 0.0067; *η*^2^ = 7.48%). An overall effect of age (*F*(1,66) = 17.71; *p* < 0.0001; *η*^2^ = 16.92%) and interaction between age and nicotine (*F*(1,66) = 14.01, *p* = 0.0004; *η*^2^ = 13.38%) was observed (*n* = 16 adult-saline, 14 adult-nicotine, 25 adolescent-saline, 28 adolescent-nicotine). **g** Nicotine exposure increased adolescent BLA microglia number (*F*(1,78) = 12.21, *p* = 0.0008; *η*^2^ = 6.44%). In addition, we found overall effect of age (*F*(1,78) = 7.987, *p* = 0.0060; *η*^2^ = 9.84%) and interaction between age and nicotine (*F*(1,78) = 26.71, *p* < 0.0001; *η*^2^ = 21.52%) (*n* = 22 adult-saline, 19 adult-nicotine, 21 adolescent-saline, 20 adolescent-nicotine). **h** Nicotine significantly decreased adult NAc IBA1 expression, but increased adolescent IBA1 NAc expression, with a significant interaction between age and nicotine (*F*(1,64) = 25.69, *p* < 0.0001; *η*^2^ = 26.96%; *n* = 10 adult-saline, 14 adult nicotine, 21 adolescent-saline, 22 adolescent-nicotine). **i** Nicotine had age-dependent effects on IBA1 BLA expression, where nicotine selectively increased IBA1 BLA expression (*F*(1,57) = 4.84, *p* = 0.0319; *η*^2^ = 6.84%), with a significant interaction between age and nicotine (*F*(1,49) = 9.629, *p* = 0.0030; *η*^2^ = 13.60%) (*n* = 15 adult-saline, 19 adult-nicotine, 13 adolescent-saline, 16 adolescent-nicotine). All data analyzed with Bonferonni post hoc analysis (*p < 0.05; **p < 0.01; ***p < 0.001; ****p < 0.0001). All *n* is number of rats. Scale bar is 50 μm. Bars show mean + /s.e.m. Source data is available as a Source Data file.

In confirmation of earlier studies from our lab^8,10^, we show that nicotine pretreatment increases initial acquisition of cocaine self-administration in adolescent rats but not adults (Fig. 1b, c). No sex differences were observed for any measure in Fig. 1, as such data were collapsed across sex.

Although it is well established that nicotine suppresses adult microglia markers^33–35^, no studies have examined the effect of nicotine on adolescent microglia. Consistent with prior findings^35^, nicotine significantly suppressed expression of the microglial marker, IBA1, in the nucleus accumbens (NAc) of adult rats (P90, Fig. 1d, h). In contrast, nicotine significantly increased IBA1 expression and IBA1 + cells in the adolescent NAc (P32, Fig. 1d, f, h). Whereas nicotine pretreatment had no significant effect on IBA1 expression in adult basolateral amygdala (BLA), there was  significant increase in IBA1 expression and IBA1 + cell count in the adolescent BLA (Fig. 1e, g, i). No other forebrain regions studied showed any nicotine-induced changes in IBA1 expression (Supplementary Fig. 1). These findings were supported with western blots (Supplementary Fig. 2). These data represent changes to microglia from nicotine alone (no cocaine), and demonstrate that nicotine has unique, age-dependent effects on IBA1 expression in the NAc and BLA, two regions that encode aspects of reward, and are actively maturing during adolescence^16,19^.

### Nicotine exposure promotes a ramified phenotype in adult microglia, and a reactive phenotype in adolescent microglia

**Fig. 2:**
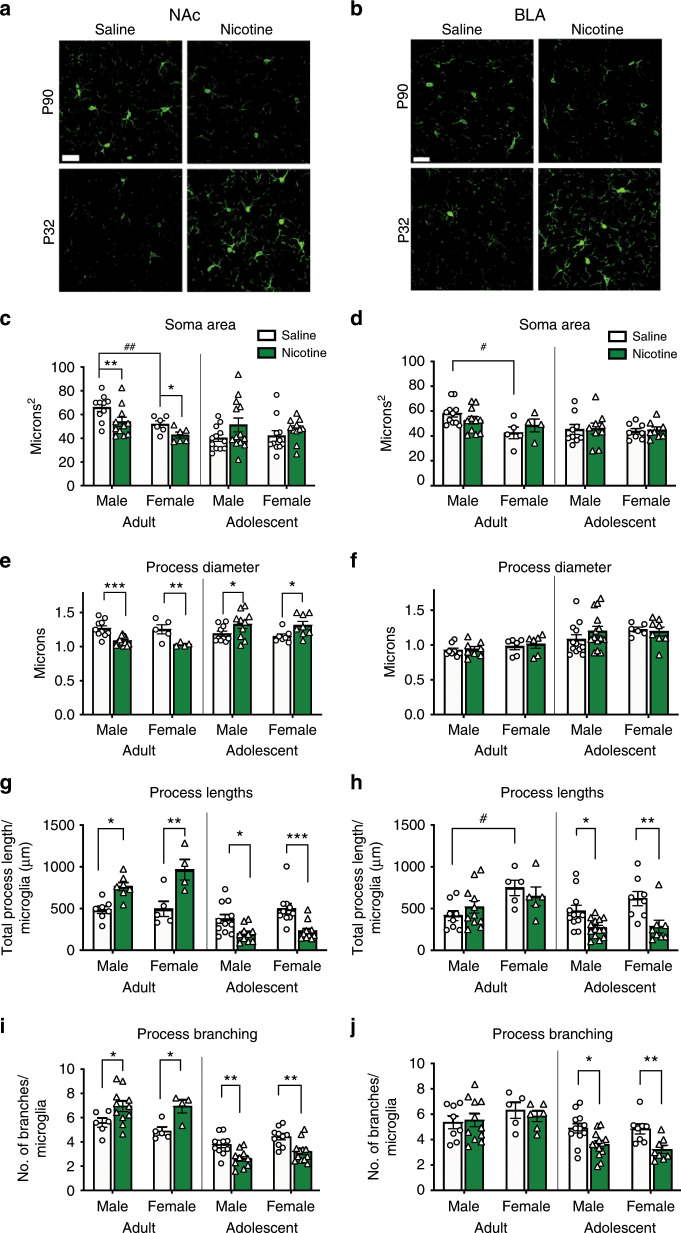
Nicotine promotes a reactive phenotype in adolescent microglia, and a ramified phenotype in adult microglia. **a**, **b** Example images of adult and adolescent NAc (**a**) and BLA (**b**). **c** In adults, soma area was signficantly affected by sex (*F*(1,29) = 14.58, *p* = 0.0007; *η*^2^ = 26.21%) and nicotine (*F*(1,29) = 16.67, *p* = 0.0003; *η*^2^ = 22.92%) (*n* = 6 adult-females, 10 adult-male-saline, 11 adult-male-nicotine). There were no significant effects of sex or nicotine on adolescent soma. **d** Adult male BLA were larger than adult-female (*F*(1,27) = 6.475, *p* = 0.0170; *η*^2^ = 17.74%; *n* = 5 adult-female-saline, 4 adult-female-nicotine, 11 males). There were no significant changes in BLA soma area following nicotine exposure at either age. **e** Nicotine significantly decreased adult NAc process diameter (*F*(1,28) = 26.14, *p* < 0.0001; *η*^2^ = 45.10%) with no sex effect (*n* = 5 adult-female-saline, adult 4 female-nicotine, 10 adult-male-saline, 13 male-nicotine) and increased adolescent NAc process diameter (*F*(1,31) = 11.59, *p* = 0.0018; *η*^2^ = 27.06%) with no effect of sex (*F*(1,31) = 0.01051, *p* = 0.9190; *η*^2^ = < 0.1%; *n* = 7 adolescent-female-saline, 8 adolescent-female-nicotine, 10 adolescent-male). **f** No significant sex or drug effects were observed in BLA process diameter (*n* = 6 adult-saline, 8 adult-male-saline, 9 adult-male-nicotine, 6 adolescent-female-saline, 12 adolescent-male-saline, 14 adolescent-male-nicotine). **g** Nicotine significantly increased (*F*(1,19) = 26.12 *p* < 0.0001; *η*^2^ = 56.33%; *n* = 5 adult-female-saline, 4 adult-female-nicotine, 7 adult-male) adult NAc microglial process lengths and decreased (*F*(1,39) = 22.59, *p* < 0.0001; *η*^2^ = 35.04%; *n* = 10 adolescent-female-saline, 12 adolescent-female-nicotine, 11 adolescent-male-saline, 10 adolescent-male-nicotine) adolescent NAc microglial process lengths, with no effect of sex. **h** Nicotine did not alter adult BLA process lengths, but adult females had larger processes (*F*(1,25) = 6.902, *p* = 0.0145; *η*^2^ = 20.90%; *n* = 5 adult-female, 8 adult-male-saline, 11 adult-male-nicotine). Nicotine decreased adolescent BLA process lengths (*F*(1,37) = 18.59, *p* = 0.0001; *η*^2^ = 32.12% *n* = 8 adolescent-female, 11 adolescent-male saline, 14 adolescent-male-nicotine). **i** Nicotine increased adult NAc branching (*F*(1,23) = 14.09, *p* = 0.0010; *η*^2^ = 36.17%; *n* = 5 adult-female-saline, 4 adult-female-nicotine, 7 adult-male-saline, 11 adult-male-nicotine) and decreased adolescent branching (*F*(1,29) = 23.29, *p* < 0.0001; 35.47%; *n* = 10 adolescent-female-saline, 11 adolescent-female-nicotine, 12 adolescent-male-saline, 10 adolescent-male-nicotine,). **j** Nicotine did not alter adult BLA branching (*n* = 5 adult-female-saline, 6 adult-female-nicotine, 8 adult-male-saline, 11 adult-male-nicotine), but decreased adolescent BLA branching (*F*(1,37) = 17.84, *p* = 0.002; *η*^2^ = 32.21%; *n* = 8 adolescent-female, 13 adolescent-male). All data analyzed with a two-way ANOVA for sex and drug, with Bonferonni post hoc analysis (* = nicotine-induced differences; *p < 0.05; **p < 0.01; ***p < 0.001; ****p < 0.0001) (# = sex-differences, #*p* < 0.05, ##*p* < 0.01. Scale bar is 50 μm. Bars show mean + / s.e.m. Source data is available as a Source Data file.

We next investigated if nicotine promotes reactive or ramified microglia by examining their morphology. In the adult NAc, nicotine exposure significantly decreased microglial soma area (Fig. 2c), decreased microglial process diameter (Fig. 2e), increased process lengths (Fig. 2g), and increased microglial branching (Fig. 2i). These morphological changes indicate an increase in a ramified microglial phenotype^29^ and replicate prior work demonstrating that nicotine suppresses microglial activation^34,35^. In the adolescent NAc, nicotine significantly increased microglial process diameter (Fig. 2e), decreased process lengths (Fig. 2g), and decreased microglial branching (Fig. 2i), consistent with a reactive phenotype^26^. In the BLA, adult nicotine exposure did not alter BLA microglial morphology (Fig. 2d–j), which is consistent with the lack of change observed in IBA1 expression (Fig. 1i). In the adolescent BLA, nicotine exposure did not alter microglial process diameter (Fig. 2f), but did significantly decrease microglial process lengths (Fig. 2h), and microglial branching (Fig. 2j). Altogether, these data demonstrate that nicotine promotes reactive microglial morphology in the adolescent NAc and BLA, while promoting a ramified microglial phenotype in the adult NAc.

We observed some adult sex differences in baseline NAc and BLA microglia morphology, in the absence of nicotine treatment. Male microglia soma are larger than female (Fig. 2c, d), consistent with recent findings^36^. However, no significant sex differences were seen in adolescent microglia (Fig. 2c–j). We also found significant age differences in soma size and process branching (Supplementary Fig. 3).

### Nicotine induces expression of anti-inflammatory transcripts in adults and pro-inflammatory transcripts in adolescents

**Fig. 3:**
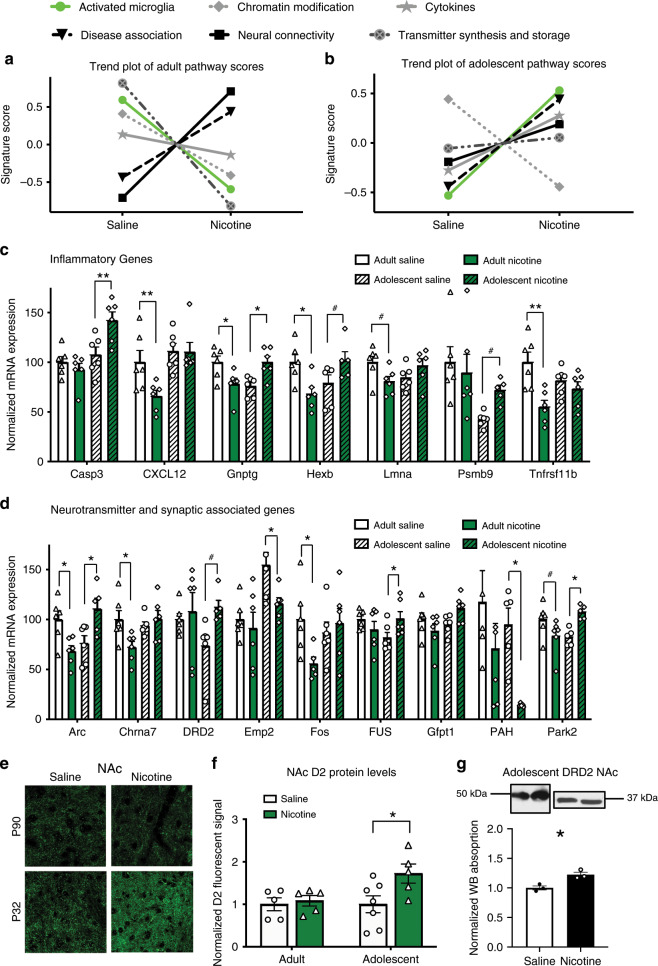
Nicotine induces age-dependent differences in gene expression. **a** Adult nicotine exposure has distinct effects on pathway scores for associated with a variety of cellular functions. In particular, nicotine decreased overall expression of genes associated with microglial activation, which is shown as a lower activated microglia pathway score. **b** Adolescent-nicotine exposure increased overall expression of genes associated with microglial activation. **c** Analysis of specific inflammatory genes, expressed as percentage of adult saline (*n* = 6) **d** Analysis of specific synaptic and neurotransmitter-associated genes, expressed as a percentage of adult saline (*n* = 6). **e** Representative images of D2 receptors in the adolescent and adult NAc. **f** Quantification of D2 receptor fluorescent intensity. A significant effect of nicotine (*F*(1,18) = 4.598, *p* = 0.0459; *η*^2^ = 16.40%; *n* = 5 adult, 7 adolescent-saline, 5 adolescent-nicotine) was observed. **g** Adolescent-nicotine exposure significantly reduced DRD2 protein levels as measured by a western blot in the NAc (*t*_4_ = 4.105, *p* = 0.0148) (*n* = 3 saline, 3 nicotine). Two-way ANOVA were further analyzed with Bonferonni post hoc tests (#*p* < .1;*p < 0.05; **p < 0.01). Bars show mean + /s.e.m. Source data is available as a Source Data file.

To further characterize nicotine-induced changes to microglia, we measured changes in messenger RNA (mRNA) transcripts associated with microglial activation in the NAc of adult and adolescent-male rats with a Nanostring multiplex neuropathology panel (Fig. 3). Activated microglial pathway scores (defined in Methods) were decreased following adult nicotine exposure (Fig. 3a), but increased following adolescent-nicotine exposure (Fig. 3b). Selected transcripts associated with microglial activation are shown in Fig. 3c. Nicotine exposure significantly decreased expression of microglial-associated transcripts *Gnptg* and *Hexb* in adults, while increasing the expression of these transcripts in adolescents. Nicotine also decreased adult expression of the cytokine receptor *Tnfrs11b*, and the inflammatory chemokine *CXCL12*, with no change in adolescents. *Casp3*, a transcript associated with apoptosis, was significantly increased following adolescent-nicotine exposure. Select transcripts were confirmed with RT-qPCR (Supplementary Fig. 4). Taken together, these data show that nicotine exposure has contrasting effects on adolescent and adult NAc immune activation-associated transcripts.

### Nicotine induces age-dependent changes in neurotransmitter and synaptic-associated transcripts

The Nanostring multiplex gene panel also measured transcripts associated with synaptic and neurotransmitter function in the adolescent and adult male NAc (Fig. 3d). Nicotine suppressed expression of the activity-related, immediate early genes *Arc* and *Fos* in adults, while increasing Arc expression in adolescents. Nicotine selectively decreased expression of the α7 nicotinic acetylcholine receptor (*Chrna7*) in adults, a receptor that underlies the suppressive effects of nicotine on adult microglia^33^. Nicotine also significantly altered transcripts associated with dopamine transmission in an age-dependent manner (Fig. 3d). Adolescent-nicotine exposure decreased expression of *PAH*, an enzyme that catalyzes the hydroxylation of phenylalanine to tyrosine.

There was also a trend for nicotine to selectively increase *DRD2* expression in adolescents. D2 receptors are functionally immature during adolescence^17,18^, and become sensitized after adolescent-nicotine pretreatment^8^. We therefore further investigated DRD2 levels with immunohistochemistry (Fig. 3e, f) and western blot data (Fig. 3g) and observed increased D2 receptor protein levels. This suggests D2 receptors may be involved in the cellular and behavioral phenomena observed, and this is further examined in the experiments below.

### Microglia are required for nicotine-induced increases in adolescent cocaine self-administration

**Fig. 4:**
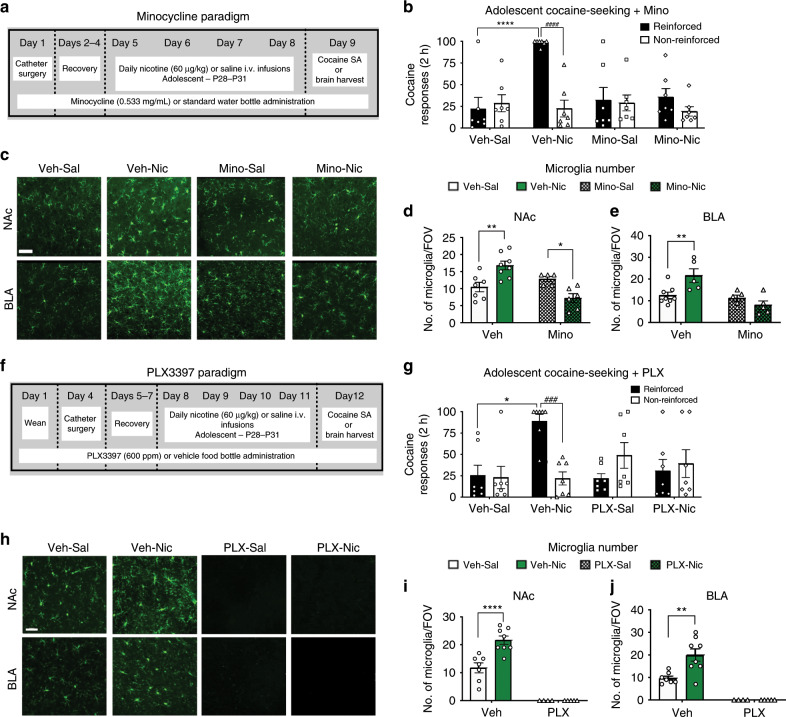
Minocycline and PLX3397 block nicotine-induced increases in cocaine seeking. **a** Schematic of paradigm with nicotine pretreatment and minocycline water bottle administration. **b** Nicotine selectively increases cocaine responding (*F*(1,24) = 26.55, *p* = < 0.0001; *η*^2^ = 12.18%) and adolescent rats discriminate between reinforced and non-reinforced responses (####*p* < 0.0001). There was a significant effect of response (*F*(1,24) = 23.03, *p* < 0.0002; *η*^2^ = 10.56%), interaction between response and minocycline (*F*(1,24) = 7.163), *p* = 0.0132, *η*^2^ = 3.286%) and interaction between response, nicotine, and minocycline exposure (*F*(1,24) = 13.97, *p* = 0.0010, *η*^2^ = 6.409%; *n* = 7). Minocycline blocked nicotine-induced increases in cocaine responding. **c** Representative images of IBA1 stained microglia in the NAc and BLA with saline or nicotine and vehicle or minocycline administration. **d** Minocycline administration eliminated nicotine-induced increases in NAc soma number (*F*(1,22) = 7.95, *p* = 0.0100; *η*^2^ = 14.14%). There was also a significant interaction between minocycline and nicotine administration (*F*(1,22) = 21.84, *p* = 0.0001; *η*^2^ = 39.66%) (*n* = 7 vehicle-saline, 5 minocycline-saline, 8 vehicle-nicotine, 6 minocycline-nicotine). **e** Minocycline administration eliminated nicotine-induced increases in BLA soma number (*F*(1,19) = 13.59, *p* = 0.0016; *η*^2^ = 32.58%). There was also a significant interaction between minocycline and nicotine administration (*F*(1,19) = 9.264, *p* = 0.0067; *η*^2^ = 22.2%) (*n* = 8 vehicle-saline, 5 minocycline-saline, 5 vehicle-nicotine, 5 minocycline-nicotine). **f** Schematic of paradigm with nicotine pretreatment and PLX3397 chow administration. **g** Nicotine selectively increases cocaine responding, with a significant interaction between response and nicotine (*F*(1,24) = 11.88, *p* = 0.0021; *η*^2^ = 8.230%). In addition, a significant interaction between responses and PLX3397 treatment (*F*(1,24) = 19.01, *p* = 0.0002; *η*^2^ = 13.17%) was observed. Adolescent rats discriminated between reinforced and non-reinforced responses (###*p* = 0.0003; *n* = 7). **h** Representative images of IBA1 stained microglia in the NAc and BLA with vehicle and PLX3397 administration. **i** Nicotine increased NAc soma number (*F*(1,20) = 10.55, *p* = 0.0040; *η*^2^ = 6.077%), and PLX3397 eliminated all observable microglia (*F*(1,20) = 119.4, *p* < 0.0001; *η*^2^ = 68.77%) (*n* = 7 vehicle-saline, 8 vehicle-nicotine, 4 PLX3397-saline, 5 PLX3397-nicotine). An interaction between nicotine and PLX3397 treatment was observed (*F*(1,20) = 10.55, *p* = 0.0040; *η*^2^ = 6.077%). **j** Nicotine increase BLA microglia soma number (*F*(1,20) = 6.388, *p* = 0.0200; *η*^2^ = 6.836%) and PLX3397 eliminated measurable BLA microglia (*F*(1,20) = 53.31, *p* < 0.0001; 57.05%). An interaction between nicotine and PLX3397 was observed (*F*(1,20) = 6.388, *p* = 0.0200; *η*^2^ = 6.836%) (*n* = 7 vehicle-saline, 8 vehicle-nicotine, 4 PLX3397-saline, 5 PLX3397-nicotine). Behavioral data were analyzed with a three-way ANOVA and immunohistochemistry data were analyzed with a two-way ANOVA. All data analyzed with Bonferroni post-hoc tests (*p < 0.05; **p < 0.01; ***p < 0.001; ****p < 0.0001). Scale bar is 50 μm. Bars show mean + / s.e.m. Source data is available as a Source Data file.

Two approaches were used to test the hypothesis that activation of microglia is necessary for nicotine-induced increase in adolescent cocaine self-administration. Minocycline, a general neuro-immune suppressor, blocked nicotine-induced enhancement of reinforced responding for cocaine (Fig. 4b). Minocycline, blocked nicotine-induced increases in microglial number (Fig. 4c–e) and activated morphology (Supplementary Fig. 5). The more selective CSF1R inhibitor, PLX3397, which clears microglia from the CNS^37^, also blocked the nicotine-induced increase in cocaine reinforcement (Fig. 4g), and eliminated microglia from the NAc and BLA (Fig. 4h–j). These findings suggest a role for microglia in the effect of adolescent-nicotine on cocaine reinforcement. Whereas it has previously been reported that PLX3397 has no negative effects on behavior^37^, this has not been studied in adolescents. In a small battery of tests, we found that PLX3397 had no significant effects on adolescent rat reward learning or stress-associated behaviors (Supplementary Fig. 6).

### D2 receptors are a critical interface between increases in microglial activation and cocaine reinforcement

**Fig. 5:**
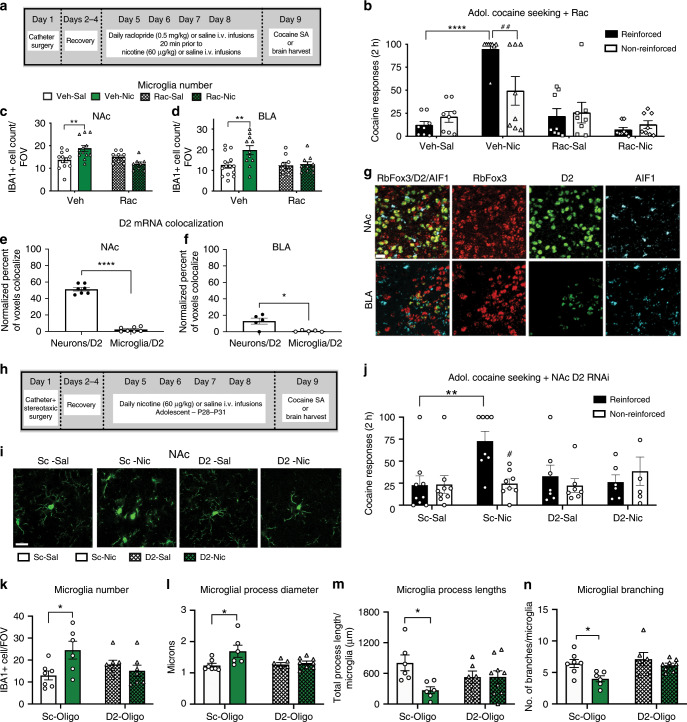
Neuronal D2 receptors are a critical interface for nicotine-induced increases to cocaine self-administration and microglia activation. **a** Schematic of paradigm. **b** Adolescent-nicotine exposure significantly increased cocaine responding (*F*(1,56) = 12.84, *p* = 0.0007; *η*^2^ = 8.977%) and raclopride blocked this (*F*(1,56) = 22.43, *p* < 0.0001; *η*^2^ = 15.68%). We also found a significant interaction between nicotine and raclopride *F*(1,56) = 35.58, *p* < 0.0001; *η*^2^ = 24.88%). Nicotine-Vehicle-treated adolescents distinguished between reinforced and non-reinforced responses (##*p* = 0.0073) (*nn* = 9 vehicle-saline, 12 vehicle-nicotine, 11 raclopride-saline, 10 raclopride nicotine). **c** Nicotine increased NAc microglia and this was blocked with raclopride (*F*(1,37) = 5.473, *p* = 0.0248, *η*^2^ = 9.597%), with a significant interaction between nicotine and raclopride (*F*(1,37) = 12.63, *p* = 0.0011; *η*^2^ = 22.15%; *n* = 12 vehicle-saline-nicotine, 9 raclopride-saline, 8 raclopride nicotine). **d** Raclopride blocked increases in BLA microglia after nicotine exposure (*F*(1,40) = 4.292, *p* = 0.0448; *η*^2^ = 7.966%; *F*(1,40) = 5.331, *p* = 0.0262; *η*^2^ = 9.893%) (*n* = 14 vehicle-saline, 11 vehicle-nicotine, 9 raclopride-saline, 10 raclopride nicotine). **e** Percent of voxels colocalized between D2 receptors, microglia (AIF1) or neurons (Rbfox3) was normalized to total D2 + voxels. Within the NAc no microglia expressed D2 receptors, while ~50% of neurons did (*t*_12_ = 20.06, *p* < 0.0001) (*n* = 7). **f** No microglia in the BLA expressed D2 receptors (*t*_8_ = 3.269, *p* = 0.0114) (*n* = 5). **g** Representative image of RNAscope colocalization of DRD2, Rbfox3 (neurons) and AIF1 (microglia) in the NAc and BLA. **h** Schematic of paradigm. **i** Adolescent-nicotine exposure s**i**gnificantly increased cocaine responding with significant interaction between nicotine, D2-RNAi and response (*F*(1,26) = 6.195, *p* = 0.0195; *η*^2^ = 8.101%). Nicotine-Vehicle-treated adolescents distinguished between reinforced and non-reinforced responses (#*p* = 0.0075) (*n* = 9 scramble-saline, 8 scrambled-nicotine, 7 D2-targeted-saline, 7 D2-targed-nicotine). **j** Representative images of IBA1 with standard and D2-targeted RNAi. **k** D2-targeted morpholino eliminates nicotine-induced increases NAc microglial count with a significant interaction between morpholino and nicotine (*F* = 1,23) = 7.2, *p* = 0.0129, *η*^2^ = 22.02%) (*n* = 7 scramble-saline, 6 scrambled-nicotine, 7 D2-targeted-saline, 7 D2-targed-nicotine). **l** D2-morpholino blocks the thickening NAc microglial process diameter (*F*(1,23) = 6.085, *p* = 0.0215, *η*^2^ = 17.42%, *n* = 7 scramble-saline, 6 scrambled-nicotine, 6 D2-targeted-saline, 8 D2-targed-nicotine). **m** D2 knockdown blocks nicotine-induced retraction of microglial processes (*F*(1,23) = 4.587, *p* = 0.0430, *η*^2^ = 14.56%) with a interaction between nicotine and morpholino (*F*(1,23) = 4.678, *p* = 0.0412, *η*^2^ = 14.85%) (*n* = 6 scrambled, 6 D2-targeted-saline, 9 D2-targed-nicotine) **n**, D2-knockdown blocks (*F*(1,23) = 4.809, *p* = 0.0387, *η*^2^ = 13.79%) nicotine-induced decreases to microglial branching (*F*(1,23) = 6.864, *p* = 0.0153, *η*^2^ = 19.69%) (*n* = 7 scramble-saline, 5 scrambled-nicotine, 6 D2-targeted-saline, 8 D2-targed-nicotine). Behavioral data analyzed with a three-way ANOVA and all morphology data analyzed with a two-way ANOVA, both Bonferonni post hoc analysis (*p < 0.05, **p < 0.01, ***p < 0.001, ****p < 0.0001). Scale bar is 50 μm. Bars show mean + /–s.e.m. Source data is available as a Source Data file.

D2 receptors are functionally immature during adolescence. Furthermore, D2 receptor density (Fig. 3e, f, g), mRNA levels (Fig. 3d), and receptor signaling^8,20^ are increased after adolescent-nicotine exposure. To investigate the role of D2 receptors in nicotine-induced increase in adolescent cocaine reinforcement and IBA1 expression, the D2 receptor antagonist, raclopride, was administered during nicotine pretreatment (Fig. 5a). D2 receptor blockade inhibited nicotine-induced increases in cocaine reinforcement (Fig. 5b).

D2 receptor activity is also required for nicotine-induced increases in microglial activation after adolescent-nicotine exposure. In the NAc and BLA, raclopride suppressed nicotine-induced increases in microglia count (Fig. 5c, d). D2 receptor blockade also significantly blocked nicotine-induced changes to microglial morphology in both the NAc and the BLA (Supplementary Fig. 7), demonstrating that D2 receptors mediate microglial activation after nicotine exposure. However, it is unclear if these D2 receptors are microglial or neuronal.

### Adolescent NAc and BLA microglia do not express D2 receptors

While our data and others demonstrate that microglia are particularly sensitive to systemic D2 receptor manipulation^38^, it is unclear if microglia express D2 receptors in vivo in the NAc or BLA. Microglia express D2 receptors in culture^39^, but recent sequencing studies of isolated microglia contradict this evidence^40^. To determine if microglia express D2 receptors, we used both immunohistochemistry and RNAscope to visualize D2 receptor colocalization on microglia and neurons. Both protein (Supplementary Fig. 8) and mRNA (Fig. 5e, f, g) analyses revealed colocalization of D2 receptors with neurons. However, both regions had little to no expression of D2 receptors on microglia.

### NAc D2 receptors are critical for nicotine induced increases in cocaine self-administration and microglial activation

To determine if NAc D2 receptors are required for nicotine-induced increases in cocaine reinforcement and microglial activation, we used a vivo-morpholino for rapid D2 RNA interference (RNAi). Microglia do not appear to express D2 receptors (Supplementary Fig. 8) and D2-targeted morpholino successfully suppressed neuronal DRD2 receptor expression (Supplementary Fig. 8b, c).

RNAi of NAc D2-receptors inhibited nicotine-induced increases in cocaine self-administration, as compared to scrambled oligo control (Fig. 5j). In addition, NAc neuronal D2-RNAi blocked nicotine-induced increases in microglial activation. Knockdown of D2 receptors blocked increases in NAc microglia number (Fig. 5k), process thickness (Fig. 5l), and decreases in process lengths (Fig. 5m) and branching (Fig. 5n). These data demonstrate that non-microglial NAc D2 receptors mediate both nicotine-induced increases in cocaine reinforcement and microglial activation.

### Fractalkine signaling mediates nicotine-induced increases in cocaine reinforcement and microglial activation

**Fig. 6:**
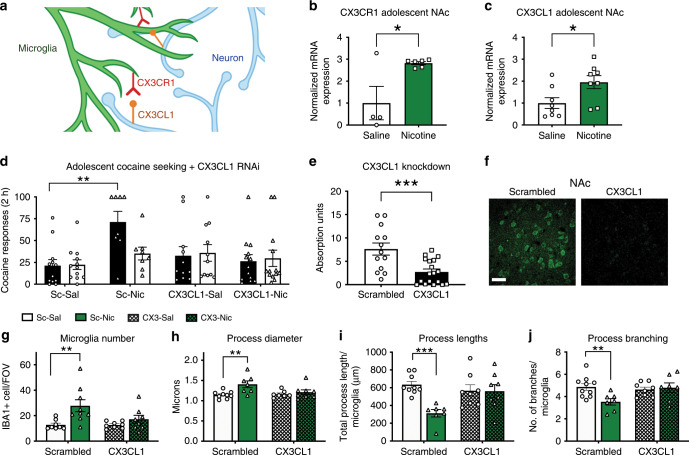
Nicotine mediates increases in cocaine self-administration and microglial activation through the CX3CL1 receptor. **a** Schematic of the microglial CX3CR1 interactions with the neuronal CX3CL1. **b** Nicotine significantly increases CX3CR1 mRNA (*t*_8_ = 2.984, *p = 0.0175, *r*^2^ = 0.5267) (*n* = 4 saline, *n* = 5 nicotine). **c** Nicotine significantly increases CX3CL1 mRNA (*t*_14_ = 2.455, *p = 0.0278, *r*^2^ = 0.3010) (*nn* = 8). **d** RNAi of CX3CL1 blocked nicotine induced increases in cocaine seeking. We observed a significant interaction between response and CX3CL1 (*F*(1, 41) = 5.450; *p* = 0.0246, *η*^2^ = 2.216), response and nicotine (*F*(1, 41) = 6.411), *p* = 0.0153, *η*^2^ = 2.606), CX3CL1 and nicotine (*F*(1, 41) = 4.530, *p* = 0.0394, *η*^2^ = 7.307), and response, CX3CL1 and nicotine (*F*(1, 41) = 4.347, *p* = 0.0433, *η*^2^ = 1.767) (*n* = 12 scrambled-saline, 8 scrambled-nicotine, 11 CX3CL1-saline, 13 CX3CL1-nicotine). **e** Knockdown verification of CX3CL1 (*t*_29_ = 3.712; *p* = 0.0009) (*n* = 13 scrambled, 18 CX3CL1). **f** Example images of CX3CL1 staining with a scrambled and CX3CL1 targeted oligo. **g** Nicotine increases microglia number in a CX3CL1-dependent manner (*F*(1, 29) = 10.02, *p* = 0.0036, *η*^2^ = 21.87) (*n* = 8). **h** Nicotine-induced increases in microglial process diameter are dependent on CX3CL1 (*F*(1, 27) = 8.578, *p* = 0.0068, *η*^2^ = 20.59) (*n* = 9 scrambled-saline, 7 scrambled-nicotine, 7 CX3CL1-saline, 8 CX3CL1-nicotine). **i** Nicotine increased microglial process lengths, and this was blocked with the CX3CL1 targeted oligo. A significant effect of nicotine (*F*(1, 28) = 8.377, *p* = 0.0073, *η*^2^ = 18.37) and interaction between RNAi and nicotine treatment (*F*(1, 28) = 7.815, *p* = 0.0093, *η*^2^ = 17.14) (*n* = 9 scrambled-saline, 7 scrambled-nicotine, 7 CX3CL1-saline, 8 CX3CL1-nicotine). **j** CXC3CL1 knockdown blocked nicotine-induced changes to microglial process branching with a significant interaction between nicotine and RNAi (*F*(1, 28) = 5.650, *p* = 0.0245, *η*^2^ = 14.46) (*n* = 10 scrambled-saline, 7 scrambled-nicotine, 8 CX3CL1-saline, 8 CX3CL1-nicotine). Behavioral data analyzed with a three-way ANOVA, morphology data was analyzed with a two-way ANOVA, and mRNA analyzed with a *t*-test. All ANOVAs further analyzed with a Bonferroni post hoc tests (*p < 0.05, **p < 0.01, ***p < 0.001, ****p < 0.0001). Scale bar 50 μm. Bars show mean + /– s.e.m. Source data is available as a Source Data file.

Fractalkine signaling, or CX3CR1-CX3CL1, is a signaling pathway that mediates microglial-neuronal communication (Fig. 6a). Both the microglial exclusive receptor, CX3CR1, and the neuronal ligand, CX3CL1, are increased in the NAc following adolescent-nicotine pretreatment (Fig. 6b, c). Increases in CX3CL1 are blocked with a DRD2 targeted morpholino, suggesting this increase is downstream of D2 receptors (Supplementary Fig. 9).

We next investigated the role of CX3CL1 in cocaine self-administration and microglial activation. Knockdown of CX3CL1 blocked nicotine-induced increases in cocaine self-administration (Fig. 6d). In addition, knockdown of CX3CL1 blocked nicotine induced increases in microglia number and activated morphology (Fig. 6g, h, i, j). Knockdown of CX3CL1 was confirmed using immunohistochemistry (Fig. 6e, f).

### Nicotine decreases synaptophysin levels in a D2-, microglia- and CX3CL1-dependent manner

**Fig. 7:**
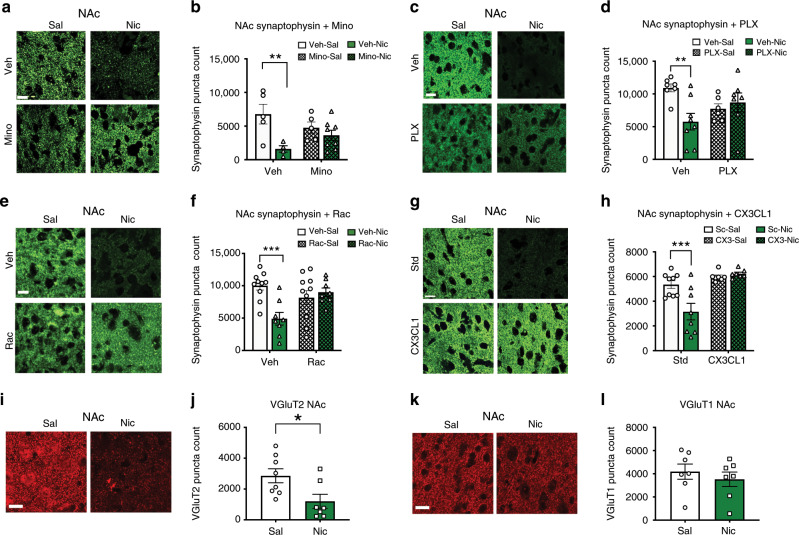
Nicotine induces a decrease in synaptic density that is microglia, D2 and CX3CL1 dependent. **a** Representative images of synaptophysin levels in the NAc following saline/nicotine and vehicle/minocycline exposure. **b** Minocycline administration prevented nicotine-induced suppression of NAc synaptophysin count (*F*(1,18) = 9.692), *p* = 0.0055, *η*^2^ = 32.59) (*n* = 5 vehicle-saline, 4 vehicle-nicotine, 5 minocycline saline, 8 minocycline nicotine). **c** Representative images of synaptophysin levels in the NAc with nicotine/saline and/or PLX3397/vehicle chow administration. **d** PLX3397 blocks nicotine induced decreases in NAc synaptophysin levels (*F*(1,24) = 7.412, *p* = 0.0119; *η*^2^ = 20.77, *n* = 7 vehicle-saline, 8 vehicle-nicotine, 6 PLX3397-saline, 7 PLX3397-nicotine). **e** Representative images of synaptophysin in the NAc with nicotine and/or raclopride administration. **f** Nicotine-induced decreases NAc synaptophysin counts (*F*(1,33) = 5.58, *p* = 0.0242; *η*^2^ = 11.18%) were blocked with raclopride (*F*(1,33) = 10.88, *p* = 0.0023; *η*^2^ = 21.81%) (*n* = 10 vehicle-saline, 8 vehicle-nicotine, 11 raclopride-saline, 8 raclopride nicotine). **g** Representative images of synaptophysin levels in the NAc following saline/nicotine and scrambled /CX3CL1 targeted oligo treatment. **h** Nicotine suppressed NAc synaptophysin levels (*F*(1, 27) = 5.889, *p* = 0.0222, *η*^2^ = 9.284), and this was blocked with CX3CL1 RNAi (*F*(1, 27) = 21.21, *p* = < 0.0001, *η*^2^ = 33.45). A significant interaction between nicotine and CX3CL1 RNAi was observed (*F*(1, 27) = 9.198, *p* = 0.0053, *η*^2^ = 14.50). **i** Representative images of Vglut2 staining in the NAc. **j** Nicotine suppresses Vglut2 levels in the NAc (*t*_13_ = 2.565, *p* = 0.0235, *r*^2^ = 0.3361) (*n* = 8 saline, 7 nicotine). **k** Representative images of Vglut1 staining in the NAc. **l** Nicotine did not significantly affect Vglut1 levels (*n* = 7). Data analyzed with a two-way ANOVA was further analyzed with Bonferroni post hoc tests (*p < 0.05, **p < 0.01, ***p < 0.001, ****p < 0.0001). Scale bar 15 μm. Bars show mean + /– s.e.m. Source data is available as a Source Data file.

Synaptic pruning is a hallmark of adolescent development, and microglia phagocytize synapses through CX3CR1 receptor signaling during development^28^. We assessed levels of the presynaptic marker, synaptophysin, using IMARIS software, and found adolescent-nicotine exposure significantly decreased synaptophysin count in the NAc (Fig. 7a–h). Nicotine-induced decreases in synaptophysin are both microglia- and D2-dependent, as they are blocked by minocycline (Fig. 7a, b), PLX3397 (Fig. 7c, d) and raclopride (Fig. 7e, f). Furthermore, CX3CL1 knockdown blocked nicotine-induced decreases in synaptophysin, implicating fractalkine signaling in this microglia-dependent phenomenon (Fig. 7g, h). In addition, nicotine decreased levels of the glutamatergic presynaptic vesicle marker VGlut2, while VGlut1 levels were unaltered by nicotine exposure (Fig. 7i–l).

## Discussion

We have shown that nicotine exposure in adolescent rats increases cocaine reinforcement, and this phenomenon reflects clinical data of teen drug use. While decades of research have correlated drug exposure with microglial activation, it was ambiguous if this activation is required for drug-seeking. We have identified microglia as a novel, yet essential, regulator of nicotine-induced increases in cocaine seeking. In addition, we found that, in the regions measured, nicotine-induced microglial activation is confined to regions that encode reward: the NAc and BLA. We found NAc D2 receptors and CX3CL1 are a mechanistic interface for nicotine-induced changes to cocaine reinforcement and microglial activation. Taken together, our data demonstrate nicotine induces unique, consequential changes to adolescent brain and behavior, and microglia are critical regulators of these permutations.

### Nicotine activates adolescent microglia, and this activation increases adolescent cocaine reinforcement

It has long been demonstrated that nicotine has distinct effects on the adolescent brain, future drug use, and other adverse behaviors. However, the mechanism by which nicotine produces these adolescent-specific perturbations has yet to be fully elucidated. Our data demonstrate a novel mechanistic role of microglia in age-dependent nicotine-induced changes to adolescent brain and behavior. We demonstrate nicotine promotes activation of adolescent microglia, while suppressing adult microglial activation. The adolescent-specific effects of nicotine on microglia are of particular clinical significance, as nearly 90% of smokers start before the age of 18^1^. Our brief, low-dose nicotine exposure paradigm mimics adolescent smoking initiation doses, and it is therefore noteworthy that we observe such profound changes in microglial function.

We also observed baseline differences in adolescent and adult microglia. Adult microglia were bigger and had more complex process morphology than adolescent microglia. Furthermore, adolescents express a different immune-associated transcriptional profile, and nicotine alters the expression of these transcripts in an age-dependent manner. These data suggest that microglia are not fully mature by adolescence and should be further characterized in this maturation period.

In addition to microglial activation, nicotine also significantly altered expression levels of transcripts associated with neuronal activity, synaptic function and dopamine transmission. Adolescent-nicotine exposure profoundly decreased expression of, *PAH*, which mediates dopamine levels in response to inflammatory cytokines^41,42^ and may contribute to the dopamine-dependent behavioral and cellular phenomena observed here.

Prior literature has demonstrated that minocycline can block rewarding aspects of drugs of abuse, and our data replicates this^30–32^. However, minocycline is a non-selective drug and it remained unclear if microglia are directly involved in reward-associated behaviors. To resolve this, we eliminated microglia in the CNS with PLX3397 and precluded increases in cocaine reinforcement. These results demonstrate the critical role of microglia in nicotine-induced increases in cocaine seeking. Notably PLX- and vehicle-treated rats did not differ in natural reward learning, suggesting that microglia may be primarily involved in drug-associated behaviors. Altogether, this highlights microglia as a promising therapeutic target for substance use disorders. The role of microglia should be studied with other drugs of abuse, as well as in other addiction-like behaviors, such as dependence and reinstatement.

### DRD2 and CX3CL1 underlie nicotine-induced changes to behavior and microglia

Neuronal D2 receptors are functionally immature during adolescence, where activation of adolescent D2 receptors increases, rather than decreases, the probability of neuronal firing^17,18^. Neurons express D2 receptors, but microglia do not. Systemically blocking D2 receptors with raclopride and suppressing NAc D2 receptors with a morpholino eliminates nicotine-induced increases in cocaine reinforcement.

We demonstrated non-microglial D2 receptors underlie increases in microglia activation after nicotine exposure. However, it remained unclear how a predominately neuronal receptor has such profound effects on microglia. We next demonstrated that the CX3CL1-CX3CR1, neuronal-microglial communication pathway is increased after adolescent-nicotine exposure. We also demonstrated CX3CL1 mediates nicotine-induced increases in microglial activation. We hypothesize the increase in CX3CL1 is downstream of D2 receptor signaling as nicotine-induced increases in CX3CL1 are blocked with a D2 knockdown. Finally, we showed nicotine-induced increases in CX3CL1 mediates nicotine-induced increases in cocaine self-administration. Altogether, these data demonstrate a multistep signaling cascade underlying microglial activation following adolescent-nicotine exposure. How D2 receptor signaling may increase CX3CL1 is unclear. One potential mechanism is through cAMP (Supplementary Fig. 10), which is decreased following D2 receptor activation and can modify CX3CL1 expression^43^. Importantly, all three elements of this signaling cascade, D2 receptors, CX3CL1 and microglial activation, are required for nicotine-induced increases in cocaine self-administration.

### D2 receptors, microglia and CX3CL1 are required for nicotine-induced decreases in synaptophysin

Synaptic pruning is a fundamental aspect of adolescent brain development and microglia prune synapses during development through CX3CR1-CX3CL1 signaling^29^. Nicotine decreased levels of the presynaptic markers synaptophysin and VGlut2, while leaving VGlut1 unchanged. VGlut1 and VGlut2 have different expression profiles throughout the brain^44^, and this may suggest nicotine has circuit-specific effects on synaptic physiology. Co-administration of minocycline, and PLX3397 blocked nicotine-induced suppression of NAc synaptophysin levels. Moreover, knocking down CX3CL1 blocked nicotine-induced decreases in synaptophysin. Taken together we show nicotine suppresses synaptophysin levels in a microglia-dependent manner through CX3CL1 signaling.

Overall, we show that adolescent-nicotine exposure promotes a maladaptive reactive microglial state, which causes an increase in cocaine reinforcement. Moreover, we demonstrate that D2 receptors and CX3CL1 are a part of a mechanistic signaling cascade that contributes to nicotine-induced changes to cocaine reinforcement, and microglial activation. Importantly, our data emphasize the exceptionally deleterious effects of nicotine on the adolescent brain. Since the introduction of e-cigarettes, teen nicotine use is increasing at an astounding rate, and over 1/3 high-school students have used e-cigarettes^5–7^. Given this, understanding how nicotine affects the adolescent brain, and identifying novel therapeutics is essential to addressing this growing teen nicotine crisis^45^. We propose microglia as a promising therapeutic target that remains understudied.

## Methods

### Subjects

Male and female Sprague–Dawley rats (Charles River) were obtained as juveniles aged P17 with dams, or as adults aged P74. Young rats, after weaning at P21, and adults were group-housed in an AALAC-accredited temperature (21 °C) and humidity (50%)-controlled vivarium, on a 12-h light–dark cycle with food and water available ad libitum. All experimental procedures were in compliance with the NIH guidelines and were approved by the Institutional Animal Care and Use Committee of the University of California, Irvine, CA.

### Surgical procedures

Before pretreatment, rats were surgically prepared with a chronic catheter implanted into the right external jugular vein^8^. Animals were anesthetized with Equithesin (0.3 ml/100 g). The catheter was passed subcutaneously from the animal’s back to the jugular vein where the tubing was inserted. The polyethylene assembly containing the opening to the catheter was mounted on the animal’s back. The cannula was flushed daily with sterile heparinized saline solution (600U heparin per 30 ml saline) to maintain patency. Animals were given 3 days to recover before beginning experiments. After experimental testing, catheter patency was evaluated with propofol. Data were discarded from animals not showing immediate anesthesia.

Morpholino intracranial injections (1.0 µl at 2 µM, total 2 pmol) were targeted at the NAc ( + 2.1AP, + /–0.5 ML, –6.9DV). Sequence for the D2-targeted morpholino: 5ʹTTCCAGCTCCCGCCGCATCCAC3ʹ. Sequence for CX3CL1 targeted morpholino: 5ʹCACGCGAGCTGTGAGGGAGCCAT3ʹ. Sequence for standard morpholino: 5ʹAAC GAA CGA ACG AAC GAA CGA ACG C3’ Injection occurred at 0.2 µl/min. Knockdown for each animal was confirmed with immunohistochemistry.

### Drugs

Cocaine was provided by the National Institute of Drug Abuse. Nicotine, raclopride, and minocycline were purchased from Sigma. All drugs were dissolved in sterile saline and filtered through sterile filters (Millipore Millex). Nicotine concentrations (pH 7.4) are expressed as nicotine base. PLX3397 chow was kindly donated by Plexxikon.

### 4-Day pretreatment

Two intravenous injections of nicotine (30 µg/kg/0.1 ml), or saline, spaced 1 min apart, were administered daily for 4 consecutive days during early adolescence (P28–31), or adulthood (P86–89; Fig. 1a). This nicotine dose yields initial blood nicotine levels similar to that produced by 1–2 cigarettes^10^ and is equivalent to typical initial smoking experiences in teens^16^. The D2 receptor antagonist raclopride (0.5 mg/kg) was administered 20 min prior to each saline or nicotine infusion (P28–P31). This dose is sufficient to block D2 receptor activity with no severe effects on behavior^37^. Minocycline was administered in the water bottle at 0.533 mg/ml from catheterization (P24) until cocaine self-administration (P32). PLX3397 was administered at 600 ppm in the chow from weaning (P21) until cocaine self-administration (P32). Vehicle chow was administered to control groups. Both chows have equivalent nutritional profiles^39^.

### Behavioral tests

For intravenous self-Administration, animals were placed into an operant chamber equipped with two nose poke holes, and were tested in a single 2 h session on a fixed ratio 1 schedule to deliver a 0.02 ml intravenous infusion of cocaine (0.5 mg/kg)^8^. To control for nonspecific activating effects of drugs, activity on a non-reinforced hole was recorded. A maximum of 100 infusions was allowed.

For open-field tests adolescent rats were habituated for 30 min prior to testing. Time spent in the center area of the apparatus was measured over 30 min using an open-field activity system measuring 43.2 × 43.2 × 30.5 cm (MED Associates). For light–dark box test adolescent rats were habituated for 10 min prior to testing. Animals were placed into the dark side of the chamber and activity was measured for 5 min.

For food training adolescent rats were food restricted to 15 g a day, beginning 3 days prior to food training. Rats were weighed every day to ensure proper growth. Food was given 15 min after each experimental session. Adolescent rats were trained to lever-press for food pellets (45 mg rodent purified diet; Bio-Serv) under a fixed ratio 1 schedule with a 1-s timeout period (FR1TO1), followed by FR1TO10, and completed with FR1TO20. Rats progressed to the next timeout period when they earned at least 30 reinforcers in the daily 30-min session.

### Immunohistochemistry and western blotting

All animals were perfused according to established procedures and brains were post fixed in 4% paraformaldehyde for 24 h. Forty micrometer coronal sections were run through free-floating immunohistochemistry (IHC). Tissue was blocked and permeated in 0.2% Triton X-100, 5% normal goat serum (NGS) in PBS for 1-2 h. Tissue is incubated with primary antibody in incubation buffer (5% NGS, 0.2% Triton X-100 in PBS) overnight. Primary antibodies used were IBA1 (1:1000; Wako-Chem; 019-1974); Synaptophysin, (1:250; Abcam; ab32127); DRD2 (1:500; Merk Millipore AB508P); GFAP (1:500; Abcam; ab4674); Neurotrace (1:300; Invitrogen); VGlut1 (1:500; Synaptic Systems, 135 304); VGlut2 (1:500 Synaptic Systems, 135 404); Fractalkine (1:250, Abcam ab25088). Tissue was incubated with secondary antibody (goat-anti-rabbit; goat-anti-chicken and donkey-anti guinea pig IgG Alexa fluor 488, 555, Life technologies) in incubation buffer. Omission of the primary antibody was a negative control.

For western blotting, 1 × 1 mm punches were taken of the NAc and BLA. Protein was isolated by homogenization on ice in tissue protein extraction reagent (T-PER; ThermoFisher Scientific) in the presence of protease and phosphatase inhibitors. The final protein concentration was determined using the Bio-Rad protein assay bovine serum albumin standards. Tissue samples were prepared in a standard 5 × SDS/PAGE sample buffer (1 m Tris, pH 6.8, 20% v/v glycerol, 10% w/v SDS, 0.05% bromophenol blue, and 10 mm 2-β-mercapto-ethanol). Ten micrograms of protein were loaded per well and run at 200 V for 50 min on NuPage 10% Bis-Tris polyacrylamide gels (Invitrogen). Electrophoretic transfer was then performed overnight at 15 V at 4 °C onto polyvinylidene difluoride membrane. Membranes were blocked for 1 h at room temperature in blocking solution (TBS Tween 20 Starting Block; ThermoFisher Scientific) and then incubated in primary antibodies (IBA1, at a 1:1000 dilution, DRD2 at a 1:1000 and synaptophysin at a 1:1000 dilution using the same antibodies as above) with agitation for 1 h at room temperature. Membranes were then rinsed three times for 5 min each in PBS with agitation. Membranes were subsequently incubated in secondary antibody (1:5000 mouse anti-rabbit HRP light chain). Supersignal Westpico Chemiluminescent substrate (ThermoFisher Scientific) was used for chemiluminescent detection according to the manufacturer’s instructions and analyzed using NIH ImageJ software. For western blot analysis, band intensity was normalized to that of GAPDH. In addition, all quantifications were normalized to control animals set at 100%. See Supplementary Fig. 11 for full blots.

### RNAscope

RNAscope version 2 kit was used (ACDBio). Animals were perfused post fixed for 72 h in 4% paraformaldehyde and sectioned at 10 μm. Manufacturer’s instructions were followed.

### Microscopy and analysis

Images for absorption analysis were captured on an Olympus Scanner BX61VS and image analysis was performed using ImageJ. A Zeiss LSM 700 was used to capture all other images. Cell morphology, count and soma size were analyzed with IMARIS. Analysis was preformed blind.

### Nanostring and quantitative real time RT-PCR

Total mRNA was extracted using an RNA Plus Universal Mini Kit (Qiagen) and was hybridized and multiplexed with NanoString probes, according to the manufacturer’s instructions. Counts for target genes were normalized to house-keeping genes (Aars, asb7, Ccdc127, Csnk2a2, Fam104a, Mto1, Supt7l, Tada2b) to account for variability in the RNA content. Pathway scores are calculated as the first principal component of the pathway genes’ normalized expression. The software will orient the scores such that increased score corresponds with increased expression in a majority of the pathway genes. A list of the genes in the neuropathology panel can be found at “https://www.nanostring.com/products/gene-expression-panels/ncounter-neuropathology-panels”

For quantitative reverse transcription PCR (RT-qPCR) complementary DNA was made from 150 ng of total RNA using the Transcriptor First Strand cDNA Synthesis kit (Roche Applied Science). Primers used were: GAPDH: Forward (CTGCACCACCAACTGCTTAG), Reverse (TGATGGCATGGACTGTGG); Hexb: Forward (TGGCAAGACGTTTTTGATGA), Reverse (ACTTTTCCACACTTCAACTACCG); Casp3: Forward (CCGACTTCCTGTATGCTTACTCTA), Reverse (CATGACCCGTCCCTTGAA); Arc: Forward (GCTGAAGCAGCAGACCTGA), Reverse (TCTGCTTTTCTTCACTGGTATGA); CHRNA7: Forward (GGCAAAATGCCTAAGTGGAC) Reverse (CTTCATGCGCAGAAACCAT). All values were normalized to Gapdh expression levels. Analysis and statistics were performed using the Roche proprietary algorithms based on the Pfaffl method.

### Sample sizes and randomization

No statistical methods were used to predetermine sample sizes. Sample sizes used were similar to those reported in previous publications^8,10^.

### Statistics

All data are expressed as mean ± s.e.m. Self-administration data were analyzed for total reinforced and non-reinforced responses by two-way ANOVA for response x pretreatment with repeated measures on responses. Raclopride, minocycline, PLX3397, D2-morpholino, and CX3-morpholino studies were analyzed by three-way ANOVA for drug x pretreatment x response. Immunohistochemistry and nanostring analyses were analyzed with a two-way ANOVA for age x nicotine pretreatment or nicotine pretreatment x raclopride exposure or nicotine pretreatment x PLX3397 exposure. Morphological analyses were analyzed with a two-way ANOVA for sex x drug. All significant main or interaction effects were further analyzed with Bonferroni post hoc comparisons.

### Reporting summary

Further information on research design is available in the Nature Research Reporting Summary linked to this article.

## Supplementary information

Supplementary Information

Peer Review File

Reporting Summary

## Data Availability

Source data underlying Figs. 1–7 and Supplementary Figs. 1–9 is available as a Source Data file. Data supporting the findings of this study are available from the corresponding author upon reasonable request.

## Source data

Source Data
