# Author Correction: Microglial activation increases cocaine self-administration following adolescent nicotine exposure

**DOI:** 10.1038/s41467-021-24307-1

**Published:** 2021-06-29

**Authors:** K. E. Linker, M. Gad, P. Tawadrous, M. Cano, K. N. Green, M. A. Wood, F. M. Leslie

**Affiliations:** 1 Department of Anatomy and Neurobiology, University of California Irvine, Irvine, CA USA; 2 Department of Neurobiology and Behavior, University of California Irvine, Irvine, CA USA; 3 Department of Pharmacology, University of California Irvine, Irvine, CA USA

**Keywords:** Glial development, Neuroimmunology, Reward

Correction to: *Nature Communications* 10.1038/s41467-019-14173-3, published online 16 January 2020.

Since the publication of this work, M. Gad has changed their name from M.G. Elabd. This has now been amended in the HTML and PDF versions of the article.

