# Supplementary material for: Microglial activation increases cocaine self-administration following adolescent nicotine exposure
Source: Nat Commun. 2020 Jan 16;11:306. doi: 10.1038/s41467-019-14173-3 (PMC6965638; doi:10.1038/s41467-019-14173-3)
Supplement: Supplementary file 1 — Supplementary Information [file 41467_2019_14173_MOESM1_ESM.pdf]

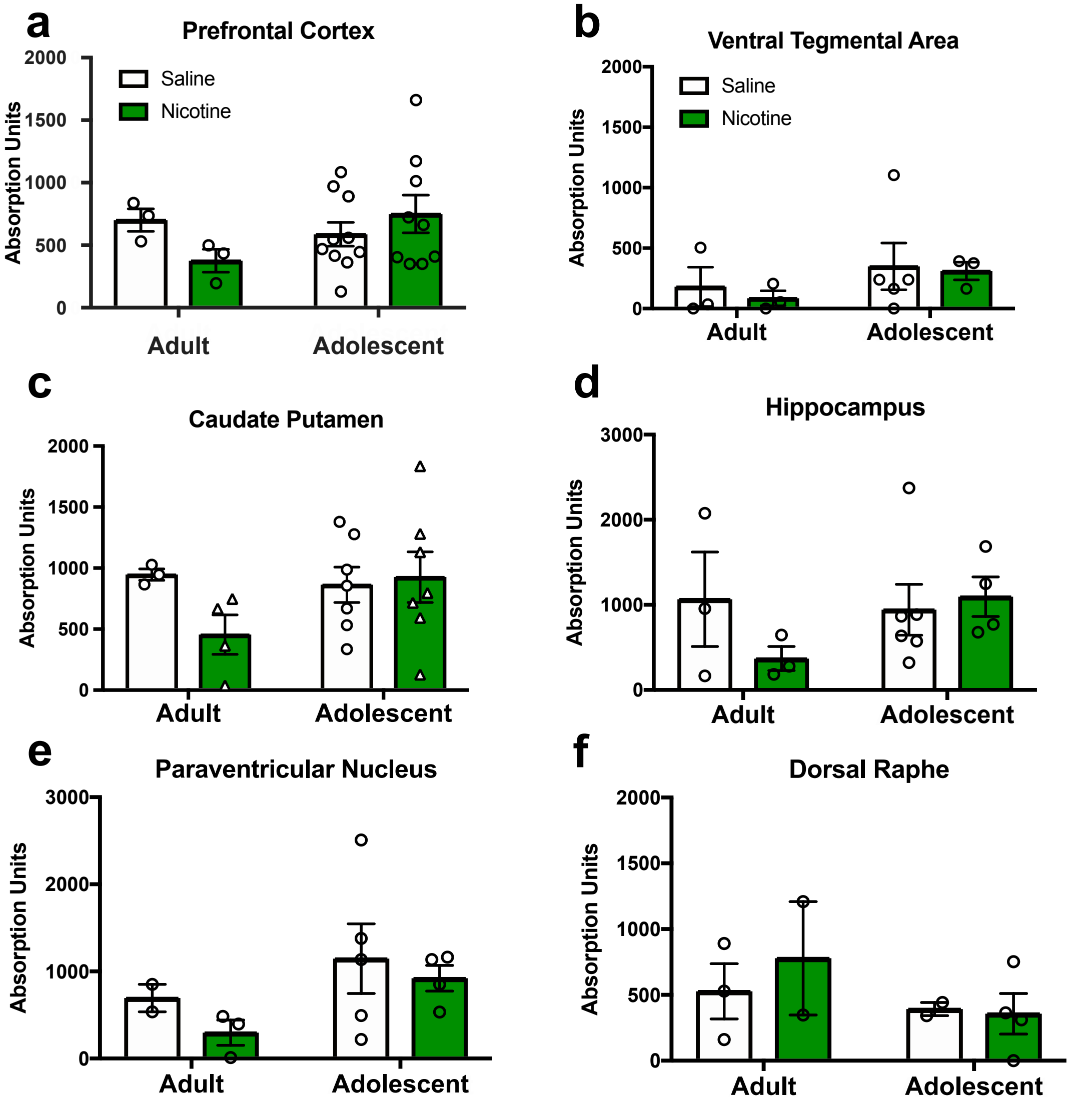

Supplementary Fig. 1: Evaluation of nicotine induced changes to IBA1 throughout the brain. **a**, Nicotine did not change PFC IBA1 expression ($F(1,21)= 0.2478$, $p=0.6238$; $\eta^2=1.305\%$; n=3-9). **b**, Nicotine did not alter VTA IBA1 expression ($F(1,10)= 0.1524$, $p=0.7044$; $\eta^2=1.312$; n=4-5). **c**, Nicotine did not significantly affect dorsal striatum IBA1 expression ($F(1,17)=1.194$, $p=0.2899$; $\eta^2=5.738$; n=3-7). **d**, Nicotine did not significantly affect hippocampal IBA1 expression ($F(1,12)=0.6186$, $p=0.4468$; $\eta^2=4.272\%$ n=3-6). **e**, Nicotine did not alter paraventricular nucleus IBA1 expression ($F(1,10)=0.8307$, $p=0.3835$; $\eta^2=5.967\%$; n=2-5). **f**, Nicotine had no effect on dorsal raphe IBA1 expression ($F(1,7)=0.2214$, $p=0.6523$; $\eta^2=2.469\%$ n=2-4). Data analyzed with a two-way ANOVA. Bars represent mean +/- s.e.m.

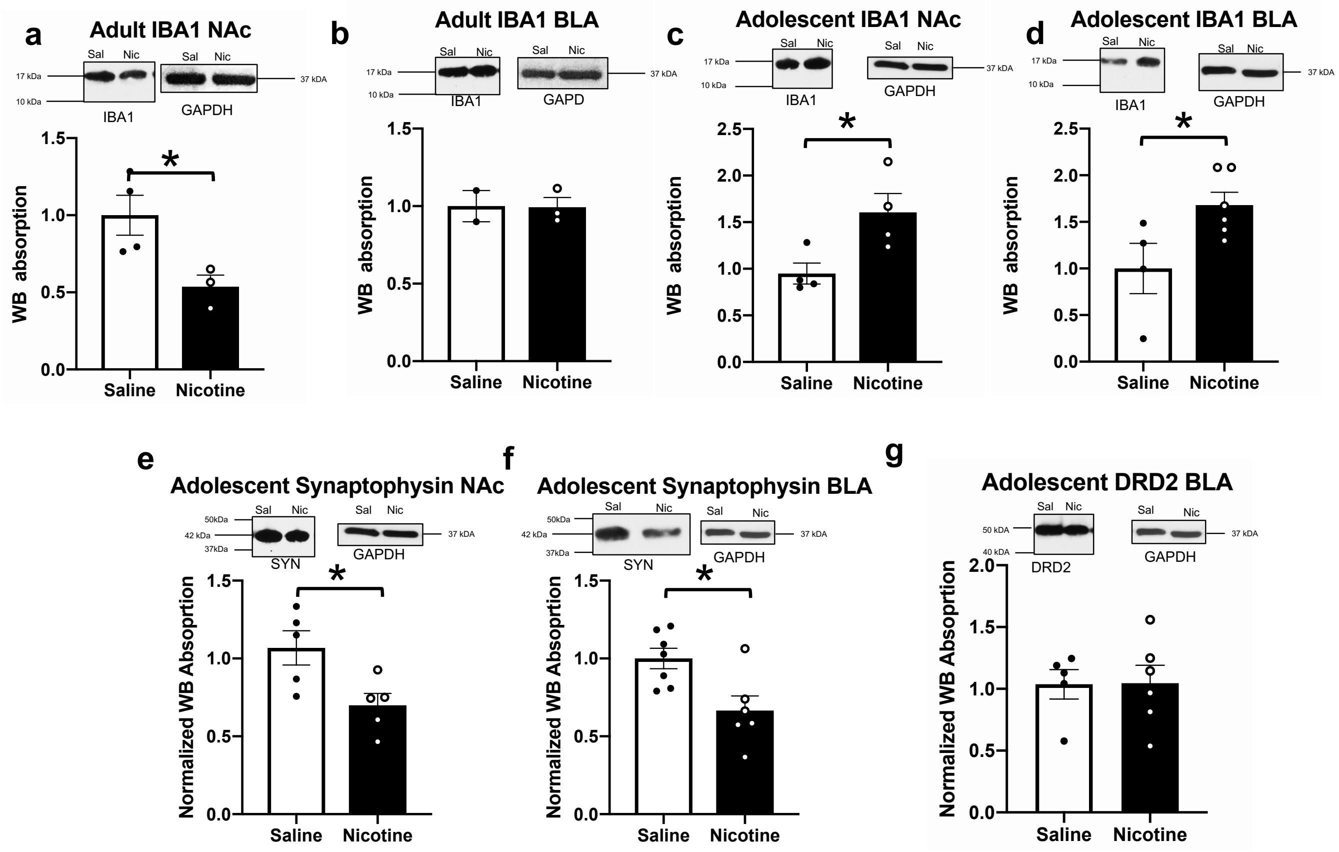

Supplementary Fig. 2: Verification of immunohistochemistry data with western blot analysis. **a,** Western blot analysis of IBA1 expression in adult NAc, showed significant decreases in IBA1 protein (t_5=2.793, p=0.0383) (n=4 saline, 3 nicotine). **b**, Nicotine did not affect adult BLA IBA1 protein levels (t_3=0.06335, p=0.9535) (n=2 saline, 3 nicotine). **c**, Adolescent nicotine exposure increased NAc IBA1 protein expression (t_6=2.834, p=0.0298) (n=4) and (**d**) BLA IBA1 protein expression (t_8=2.477, p=0.0383) (n=4 saline, 6 nicotine). **e**, Adolescent nicotine exposure decreased NAc synaptophysin protein (t_8=2.749, p=0.0251) (n=5) and (**f**) BLA synaptophysin protein (t_{11}=2.989, p=0.0123) (n=7 saline, 6 nicotine). **g**, Adolescent nicotine exposure did not alter BLA DRD2 protein levels BLA (t_9=0.0448, p=0.9652) (n=5 saline, 6 nicotine). Data analyzed with a t-test (*p<0.05, **p<0.01, ***p<0.001, ****p<0.0001). Bars show mean +/- s.e.m.

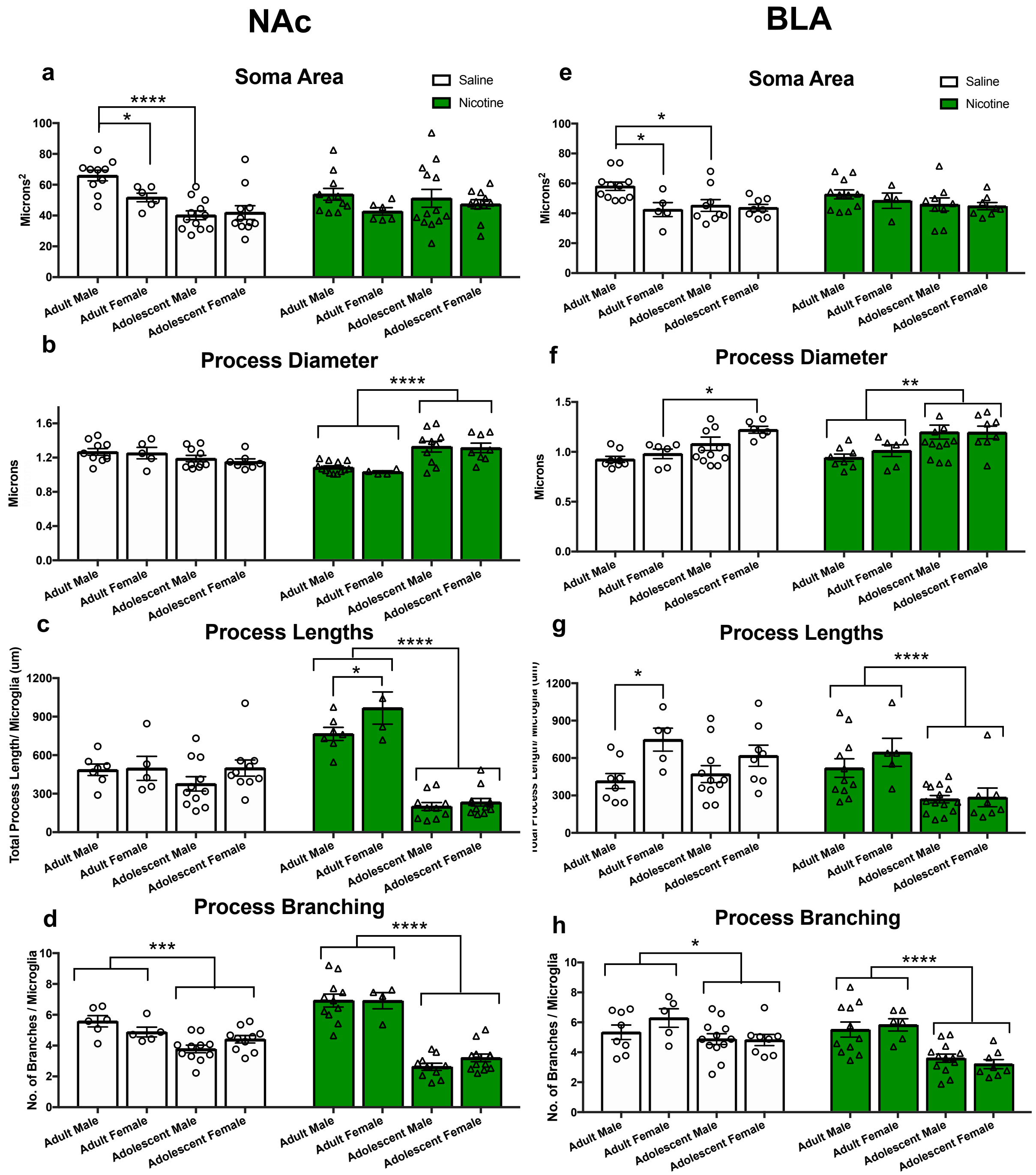

NAc
BLA
a
Soma Area
Saline
Nicotine
Microns^2
Adult Male
Adult Female
Adolescent Male
Adolescent Female
b
Process Diameter
Microns
c
Process Lengths
Total Process Length/ Microglia (um)
d
Process Branching
No. of Branches / Microglia
e
f
g
h

Supplementary Fig. 3: Comparison of adult and adolescent microglial morphology **a**, Saline treated adolescent rats and adult female rats had significantly smaller NAc soma compared to adult male rats ($F(1,35)=22.04$, $p<0.0001$, $\eta^2=32.07\%$), and there was an interaction between age and sex ($F(1,35)=4.406$, $p=0.431$; $\eta^2=6.409\%$). Nicotine had no differential effect on soma size in either age or sex (n=6-13). **b**, Baseline NAc process diameter was not significantly different in either age or sex. Nicotine significantly increased adolescent rat process diameter as compared to adult ($F(1,31)=26.81$, $p<0.0001$; $\eta^2=44.19\%$) (n=4-14). **c**, Baseline NAc process lengths were consistent across age and sex. Nicotine exposed female rats had longer process lengths compared to male ($F(1,29) = 5.567$, $p=0.252$; $\eta^2=2.79\%$), and nicotine treated adolescent rats had short process lengths than adult ($F(1,29)=170.3$, $p<0.0001$; $\eta^2=85.34\%$) (n=4-14). **d**, Saline treated adults had more NAc process branching than adolescent ($F(1,28)=14.43$, $p=0.0007$, $\eta^2=29.41\%$) and there was a significant interaction between age and sex ($F(1,28)= 4.956$, $p=0.0342$, $\eta^2=10.1\%$). Nicotine treated adolescent rats had lower branching than adult ($F(1,33)=112.6$, $p<0.0001$; $\eta^2=68.33\%$) (n=4-14). **e,** Saline treated adolescent and adult female BLA microglia were smaller than adult male microglia ($F(1,29)=6.076$, $p=0.199$, $\eta^2=13.5\%$), and there was a significant interaction between age and sex ($F(1,29)=4.096$, $p=0.0523$; $\eta^2=9.1\%$). **f**, Adolescent rats had larger baseline process diameters in the BLA as compared to males ($F(1,28)=10.91$, $p=0.0026$, $\eta^2=26.73\%$). Nicotine treated adolescent rats had thicker processes than adults ($F(1,32)= 9.838$, $p=0.0037$; $\eta^2=22.77\%$) (n=6-14) **g**, Adult female saline treated rats had longer BLA process lengths than male ($F(1,28)=9.297$, $p=0.0050$; $\eta^2=24.7\%$). Adult nicotine rats had longer process lengths than adolescent ($F(1,34)=19.48$, $p<0.0001$; $\eta^2=35.81\%$) (n=6-14). **h**, Adolescent rats had lower baseline BLA branching ($F(1,29)=4.376$, $p=0.0453$; $\eta^2=12.75\%$). Nicotine treated adolescents had higher branching compared to adults ($F(1,34)=30.6$, $p<0.0001$; $\eta^2=46.96\%$) (n=6-14). Data analyzed with a two-way ANOVA and subsequently analyzed with Bonferroni post hoc analysis (*p<0.05, **p<0.01, ***p<0.0001, ****p<0.00001). Bars show mean +/- s.e.m.

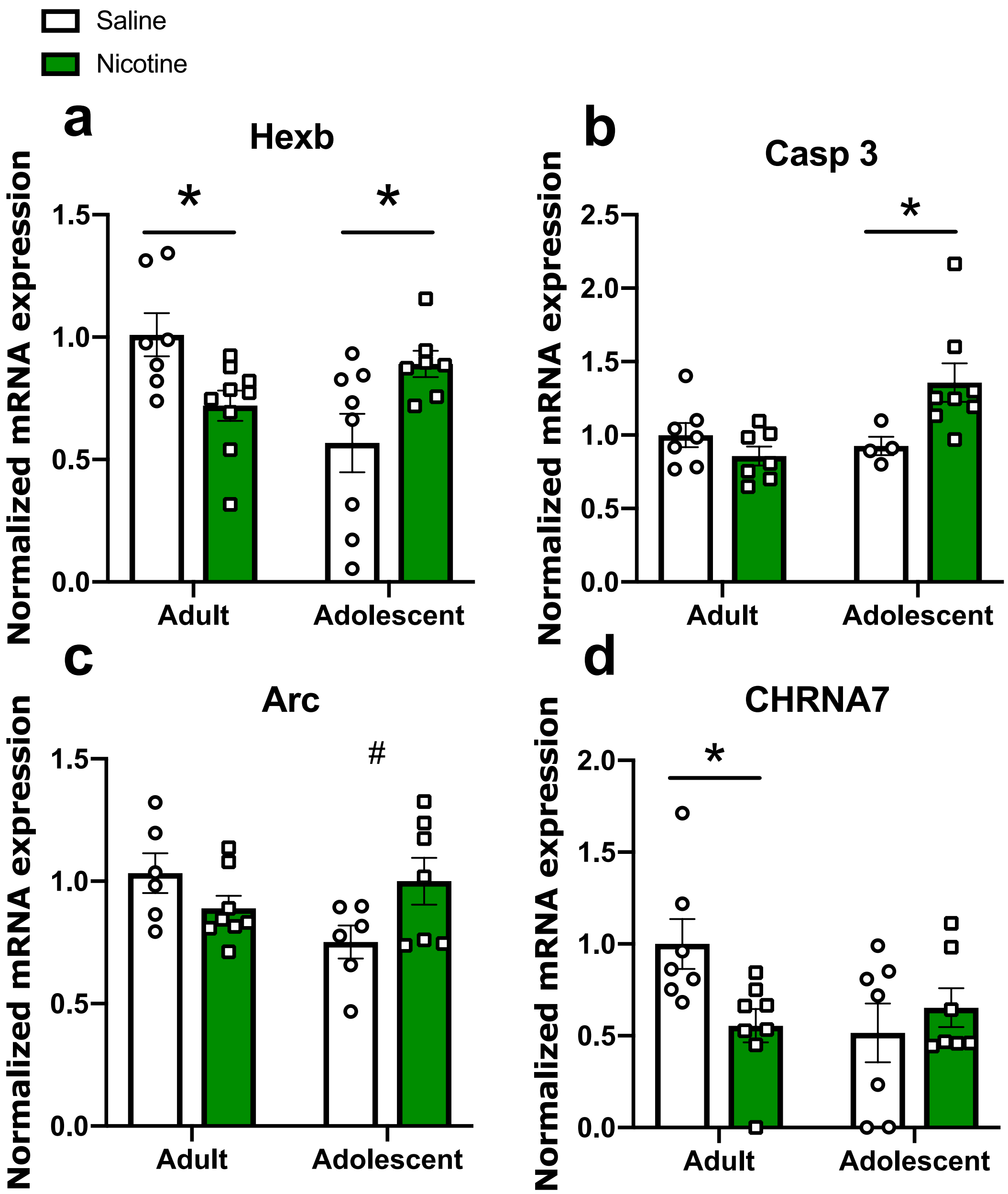

Supplementary Fig. 4: Verification of select Nanostring differentially expressed genes within the NAc with qPCR analysis. **a**, Nicotine significantly decreased adult Hexb expression, but increased adolescent ($F (1, 27)=12.71$, $p=0.0014$) (n=7 adult saline, 9 adult nicotine, 8 adolescent saline, 7 adolescent nicotine). **b**, Nicotine significantly increased adolescent Casp3 mRNA ($F (1, 22)=7.459$, $p=0.0122$) (n=7 adult saline, 7 adult nicotine, 4 adolescent saline, 8 adolescent nicotine). **c**, Nicotine increased adolescent Arc mRNA expression with a significant interaction between age and nicotine pretreatment ($F (1, 23)=6.774$, $p=0.0159$) (n=6 adult saline, 8 adult nicotine, 6 adolescent saline, 7 adolescent nicotine). **d**, Nicotine significantly decreased adult CHRNA7 mRNA expression ($F (1, 25)=5.507$, $p=0.0272$) (n=7 adult saline, 8 adult nicotine, 7 adolescent saline, 7 adolescent nicotine). Data analyzed with a two-way ANOVA and Bonferroni post hoc tests (*$p<0.05$, **$p<0.01$; #$p=0.0606$). Bars show mean +/- s.e.m.

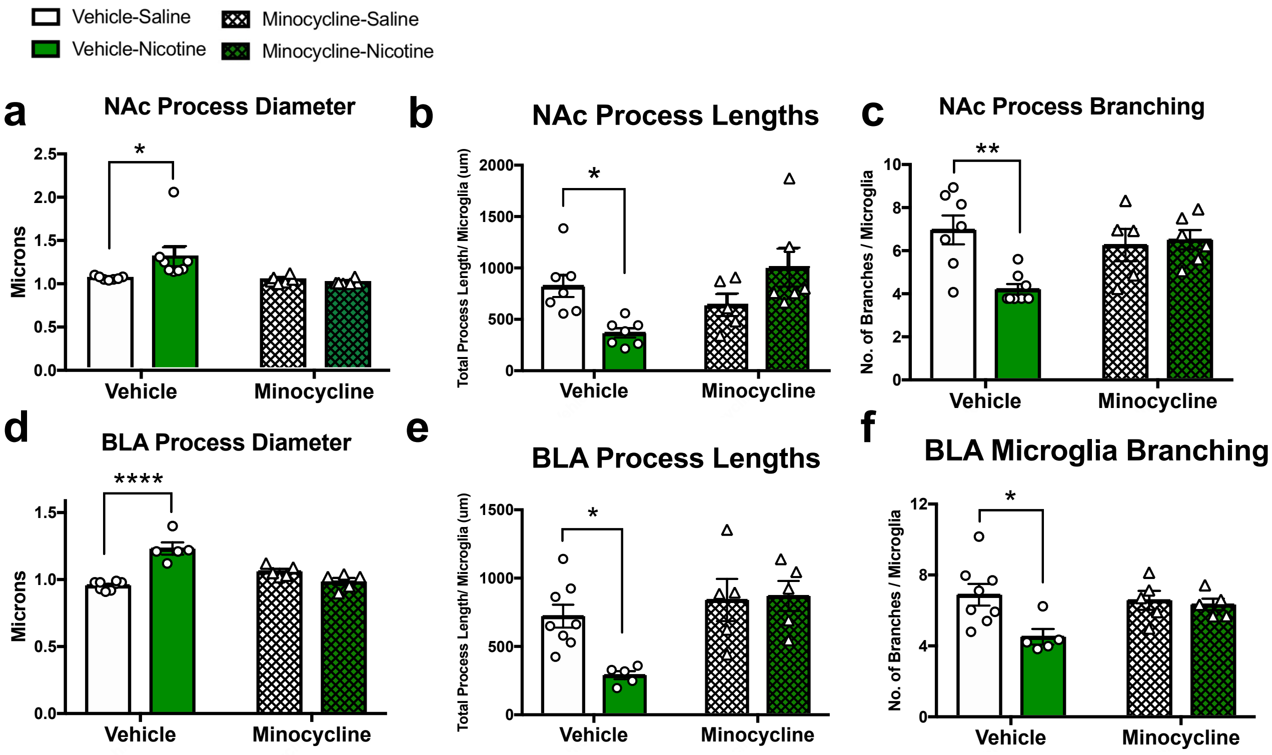

Supplementary Fig. 5: Minocycline blocks nicotine-induced microglia activation. **a**, Nicotine increased NAc diameter (F(1,23)=4.299, p=0.0495; η^2=14.8%), and this was blocked with minocycline (F(1,23)=5.447, p=0.0287; η^2=11.68%) (n=6-8). **b**, Nicotine decreased NAc process lengths, and minocycline blocked this increase (F(1,21)=11.49, p=0.0028; η^2=30.87) (n=5-7). **c**, Nicotine decreased NAc microglial branching (F(1,22)=5.532, p=0.0280; η^2=13.57%), and minocycline blocked this decrease (F(1,22)=7.935, p=0.0100; η^2=19.46%) (n=5-8). **d**, Nicotine increased BLA microglial diameter (F(1,19)=14.75, p=0.0011; η^2=16.73%) and minocycline blocked this increase (F(1,19)=7.493, p=0.0131; η^2=8.499%). There was an interaction between nicotine and minocycline (F(1,19)=45.97, p<0.0001; η^2=52.15%) (n=5-8). **e**, Minocycline blocked nicotine-induced decreases in BLA process lengths (F(1,19)=11.31, p=0.0033; η^2=30.4%). There was an interaction between nicotine and minocycline (F(1,19)=4.937, p=0.0386; η^2=13.26%) (n=5-8). **f**, Nicotine decreased BLA process branching (F(1,19)=5.446, p=0.0307; η^2=18.21%) and this blocked with minocycline administration (n=5-8). Data analyzed with a two-way ANOVA and subsequently analyzed with Bonferroni post hoc analysis (*p<0.05, **p<0.01, ***p<0.0001, ****p<0.00001). Bars show mean +/- s.e.m.

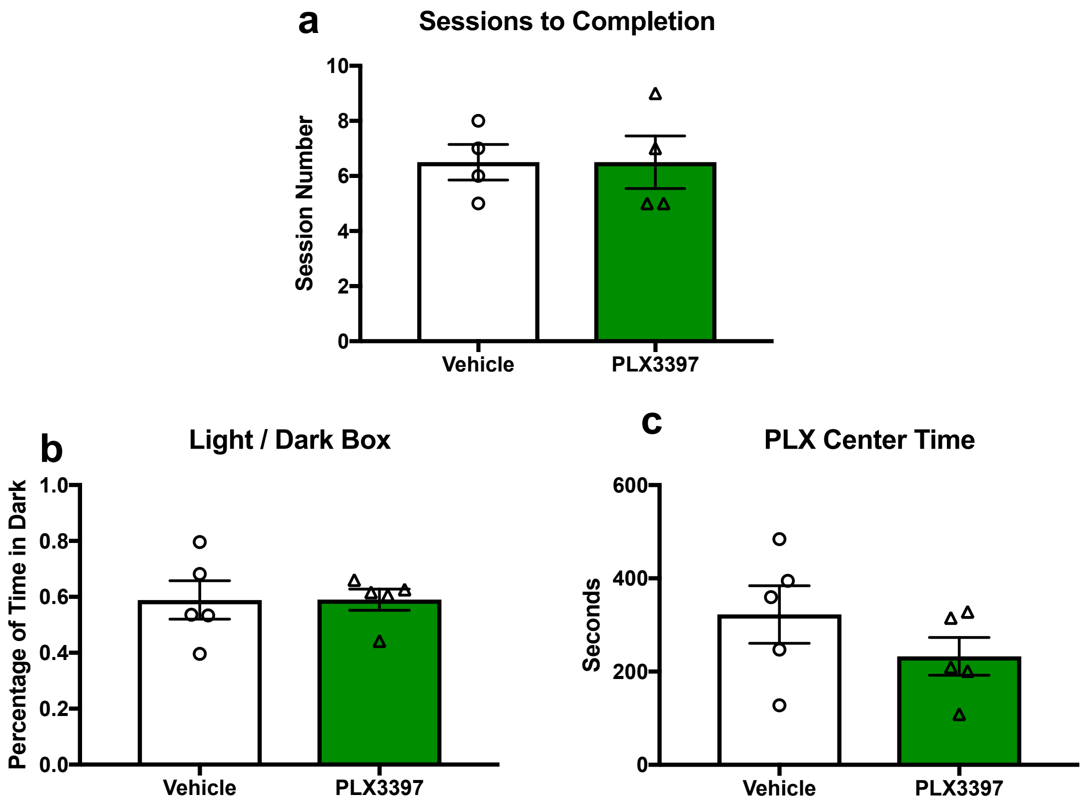

Supplementary Fig. 6: PLX3397 does not affect reward or stress behaviors. PLX3397 was administered at least 7 days prior to each behavioral test to ensure clearance of microglia (see Fig. 4). PLX3397 did not alter stress or reward behaviors measured. **a,** PLX3397 chow administration did alter food training acquisition. PLX3397 did not alter number of sessions to completion of the food-training paradigm (t_6=0, p>0.9999; n=4; r^2=0). **b**, PLX3397 did not affect light dark box performance (t_8=0.01526, p=0.9882; n=5; r^2=2.912e^{-005}). **c**, PLX3397 did not significantly change rat center time t_8=1.22, p=0.2573; n=5; r^2=1.568). Data analyzed with t-tests.

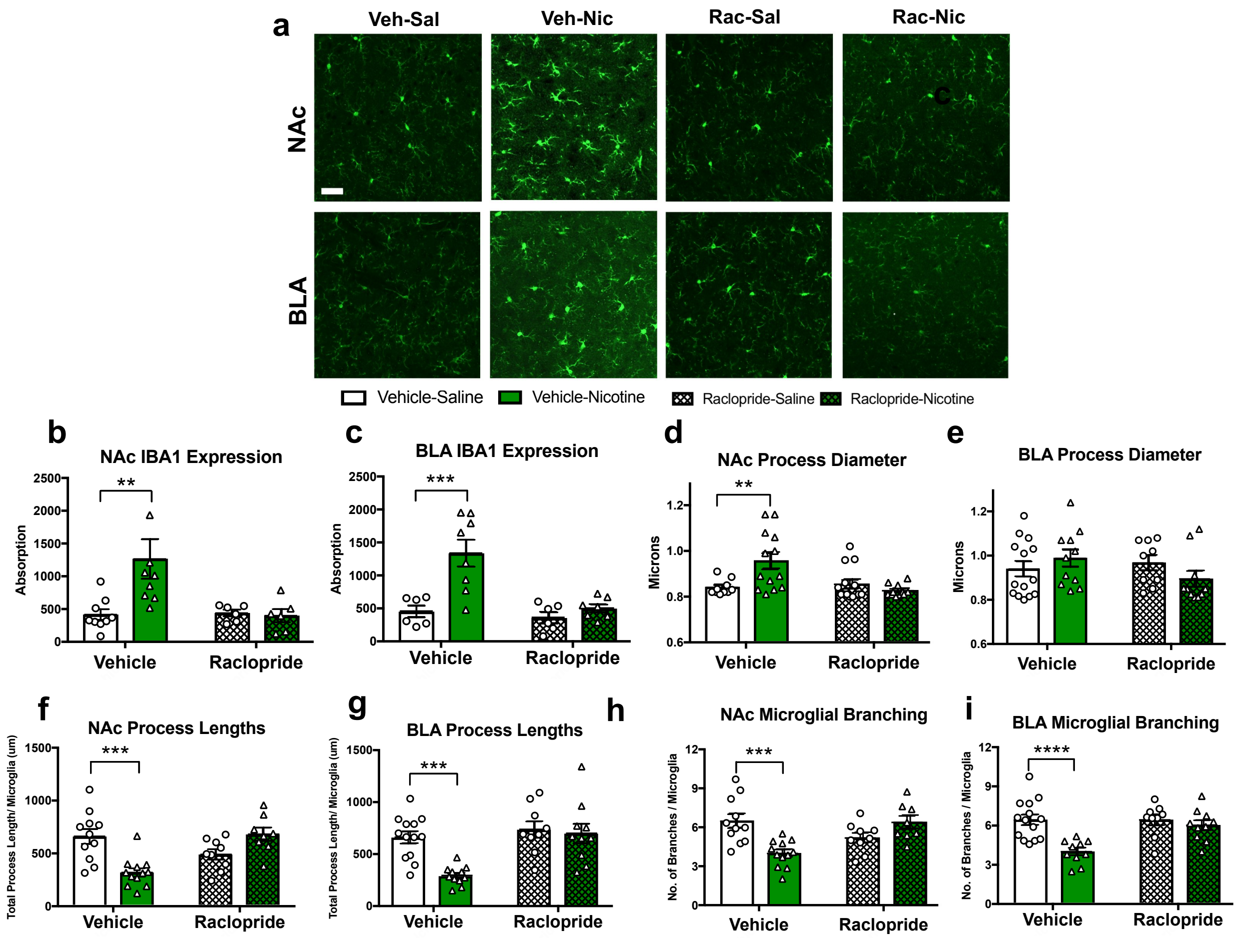

Supplementary Fig. 7: Raclopride blocks nicotine-induced microglial activation. **a,** Representative image of IBA1 stained cells with +/-nicotine and +/- raclopride. **b**, Nicotine significantly increased NAc IBA1 expression ($F(1,26)=4.154$, $p=0.0518$; $\eta^2=9.914\%$) and this was blocked with raclopride administration ($F(1,26)=4.479$, $p=0.0440$; $\eta^2=10.62\%$). We observed a significant interaction between nicotine and raclopride ($F(1,26)=4.991$, $p=0.0343$; $\eta^2=11.91\%$) (n=9 vehicle-saline/nicotine, 6 raclopride-saline/nicotine). **c**, Nicotine significantly increased BLA IBA1 expression ($F(1,23)=13.79$, $p=0.0011$; $\eta^2=23.32\%$) and raclopride eliminated this increase ($F(1,23)=7.185$, $p=0.0134$; $\eta^2=19.25\%$) (n=6 vehicle-saline, 8 vehicle-nicotine, 6 raclopride-saline, 7 raclopride-nicotine).**d**, Raclopride blocks nicotine-induced increases adolescent NAc process diameter ($F(1,44)=6.051$, $p=0.0179$; $\eta^2=9.473\%$). A significant interaction between raclopride and nicotine ($F(1,44)=9.377$, $p=0.0037$; $\eta^2=14.68\%$) was found (n=11 vehicle-saline, 14 vehicle-nicotine, 14 raclopride-saline, 11 raclopride-nicotine). **e**, No significant effects of raclopride or nicotine were found in the BLA process diameter (n= 14 vehicle-saline, 11 vehicle-nicotine, 9 raclopride-saline, 10 raclopride-nicotine). **f**, Nicotine exposure decreased adolescent NAc microglial process lengths and raclopride blocked this decrease ($F(1,36)=21.54$, $p<0.0001$; $\eta^2=33.35\%$; n= 11 vehicle-saline, 12 vehicle-nicotine, 9 raclopride-saline, 8 raclopride-nicotine). **g**, Raclopride blocks nicotine-induced decreases adolescent BLA microglial process lengths. We observed a significant effect of nicotine ($F(1,39)=9.432$, $p=0.0039$; $\eta^2=13.84$), raclopride ($F(1,39)=13.19$, $p=0.0008$; $\eta^2=19.35\%$) and interaction between nicotine and raclopride ($F(1,39)=6.24$,$p=0.0168$; $\eta^2=9.155$) (n=13 vehicle-saline, 11 vehicle-nicotine, 9 raclopride-saline, 10 raclopride-nicotine). **h,** Raclopride blocked nicotine-induced decreases in NAc microglial branching ($F(1,36)=17.47$, $p=0.0002$, $\eta^2=29.31$; n= 11 vehicle-saline, 12 vehicle-nicotine, 9 raclopride-saline, 8 raclopride-nicotine). **i**, Raclopride blocked decreases adolescent BLA microglial branching after nicotine exposure. There was a significant effect of nicotine ($F(1,39)=13.08$, $p=0.0008$; $\eta^2=13.84$), raclopride ($F(1,39)=6,564$, $p=0.0144$; $\eta^2=19.35$), and interaction between nicotine and raclopride ($F(1,39)=6.609$, $p=0.0141$; $\eta^2=9.155$) (n= 14 vehicle-saline, 10 vehicle-nicotine, 9 raclopride-saline, 10 raclopride-nicotine). All data analyzed with a two-way ANOVA, both Bonferonni post hoc analysis (*$p<0.05$, **$p<0.01$, ***$p<0.001$, ****$p<0.0001$). Scale bar is 50µm. Bars show mean +/- s.e.m.

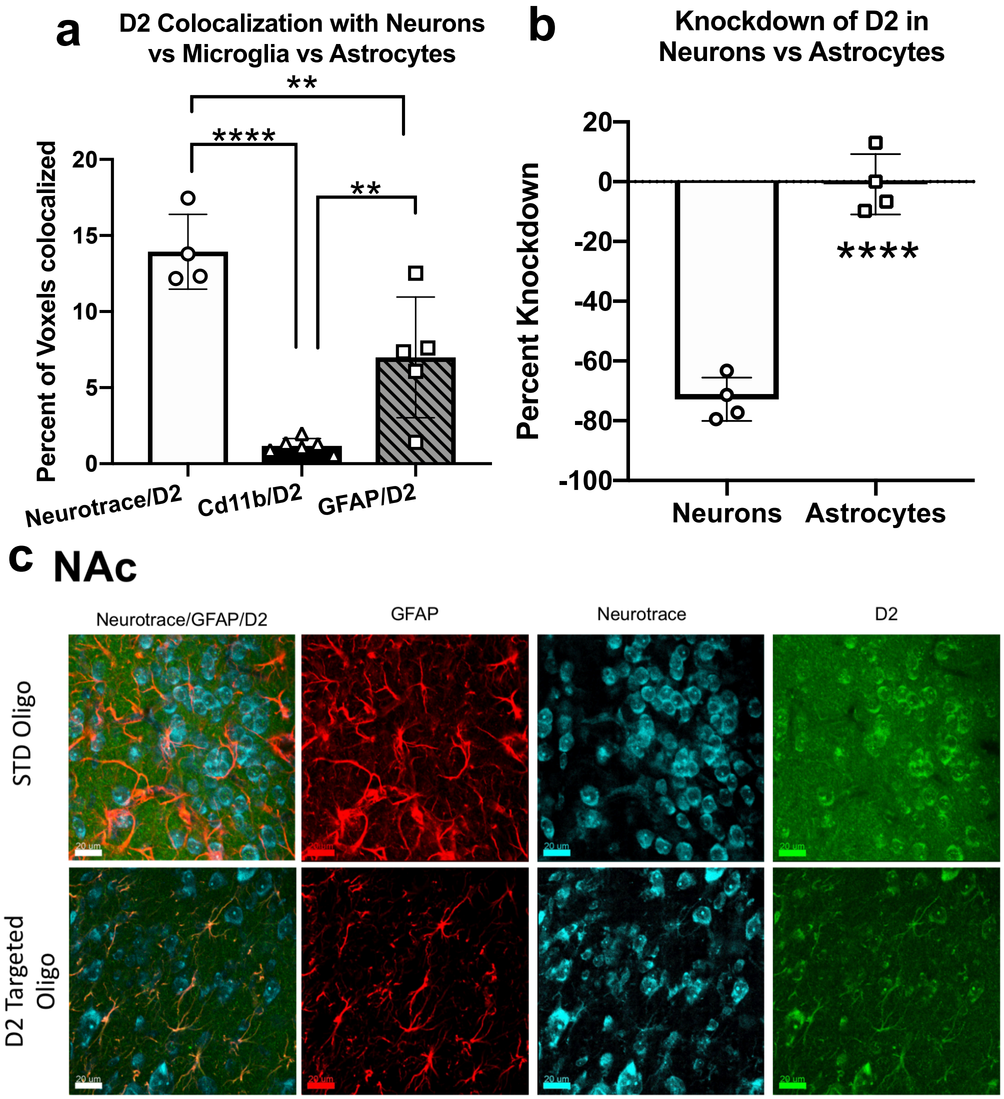

Supplementary Fig. 8: Percent knockdown of D2 receptors with morpholino was predominately restricted to neurons. **a**, Percent knockdown determined by (1-Percent decrease in voxels colocalized) compared to sc-oligo treatment. Decrease in voxels colocalized was significantly higher in neurons compared to astrocytes (t_6=11.59, p<0.0001, n=4) **b**, Representative immunohistochemistry images of colocalization between Neurotrace, GFAP, and D2. Data analyzed with a two-way ANOVA and Bonferroni post hoc tests (*p<0.05, **p<0.01, ***p<0.001, ****p<0.0001). **c**, Representative images of immunohistochemistry data shown in panel a. D2 targeted morpholino preferentially depletes D2 receptors in neurons. Scale bar is 20 µm. Bars show mean +/- s.e.m.

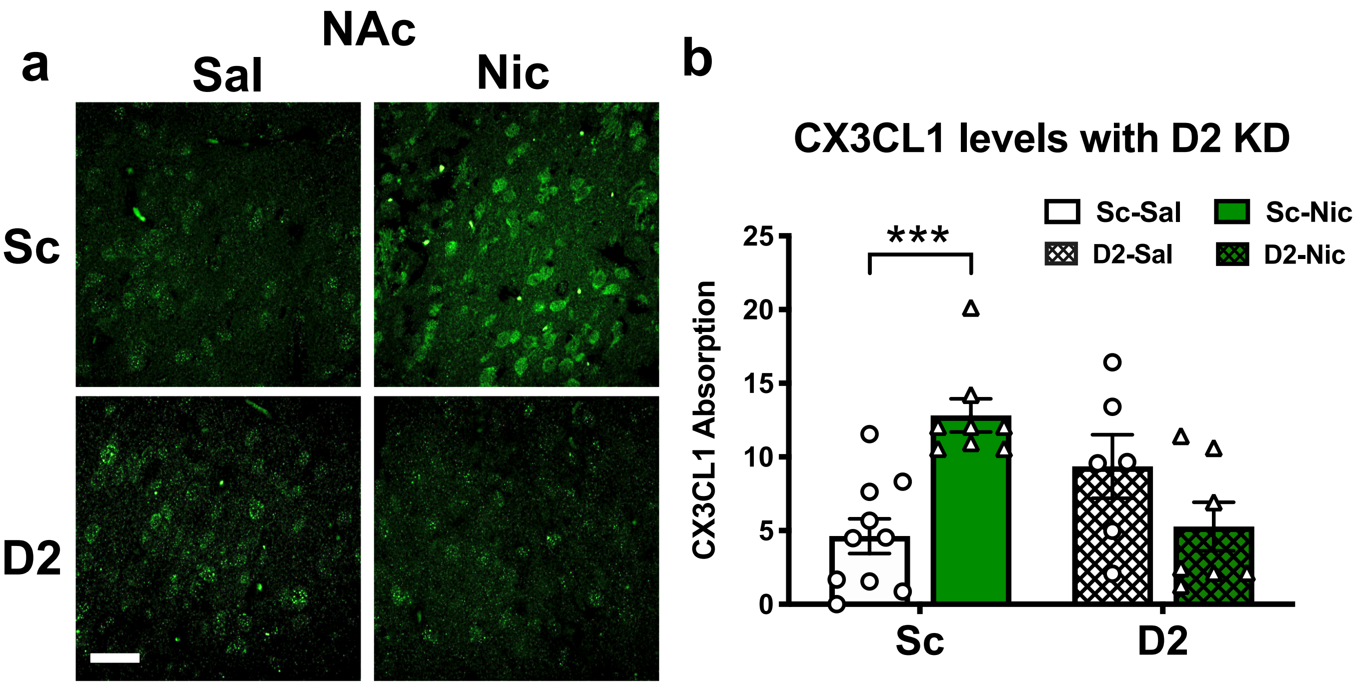

Supplementary Fig. 9: Nicotine increases CX3CL1 levels and this is blocked by DRD2 targeted morpholino. **a**, Representative images of CX3CL1 in the NAc with and without nicotine and with a scrambled or DRD2 targeted morpholino. **b**, CX3CL1 is increased after nicotine exposure, and this is blocked with DRD2 knockdown, a significant interaction between CX3CL1 and nicotine (F (1, 27) = 16.98, p=0.0003, η^2=34.80, n=6-10). Data analyzed with a two-way ANOVA and Bonferroni post hoc tests (****p<0.0001). Scale bar is 20 µm. Bars show mean +/- s.e.m.

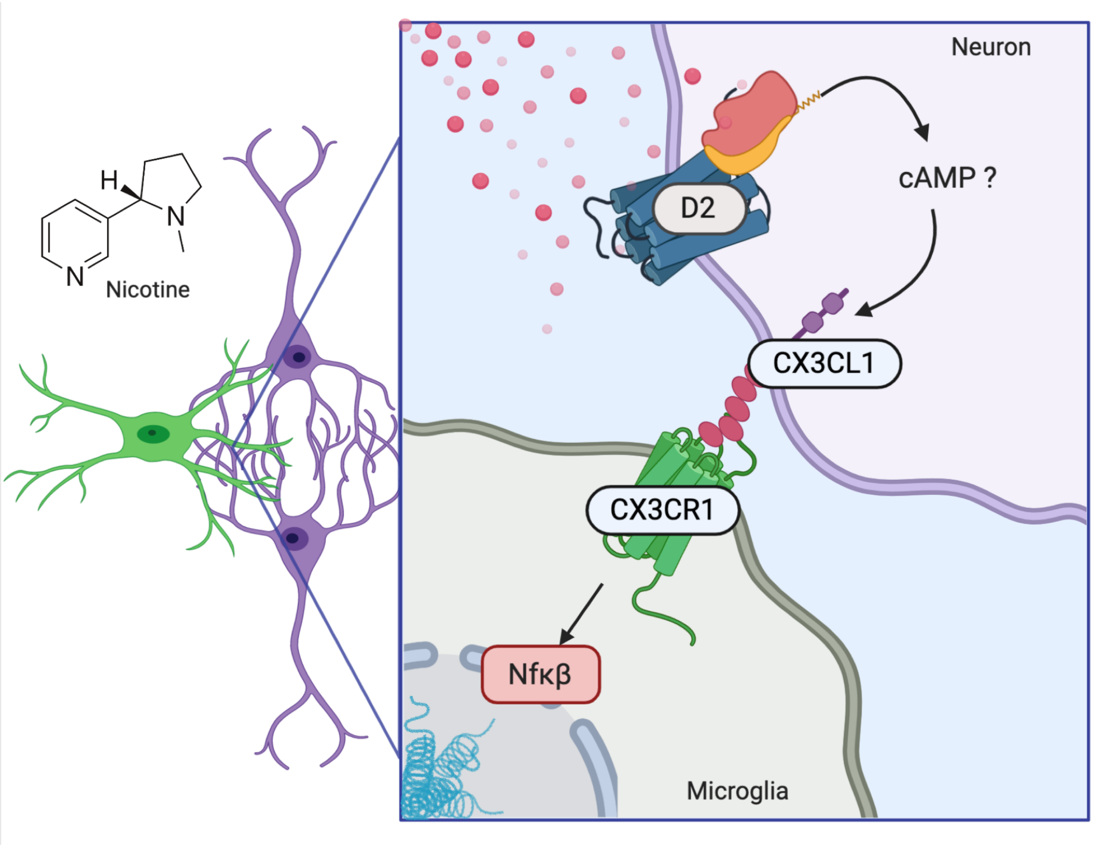

Supplementary Fig. 10: Schematic of proposed mechanism for nicotine-induced changes to adolescent microglia. Adolescents express functionally immature dopamine receptors that increase in expression after nicotine exposure. Nicotine generates a cascade of signaling events, to increase CX3CL1 via neuronal D2 receptors. CXC3CL1 binds to the microglia exclusive receptor CX3CR1 to induce microglial activation.

IBA1 Western Blot

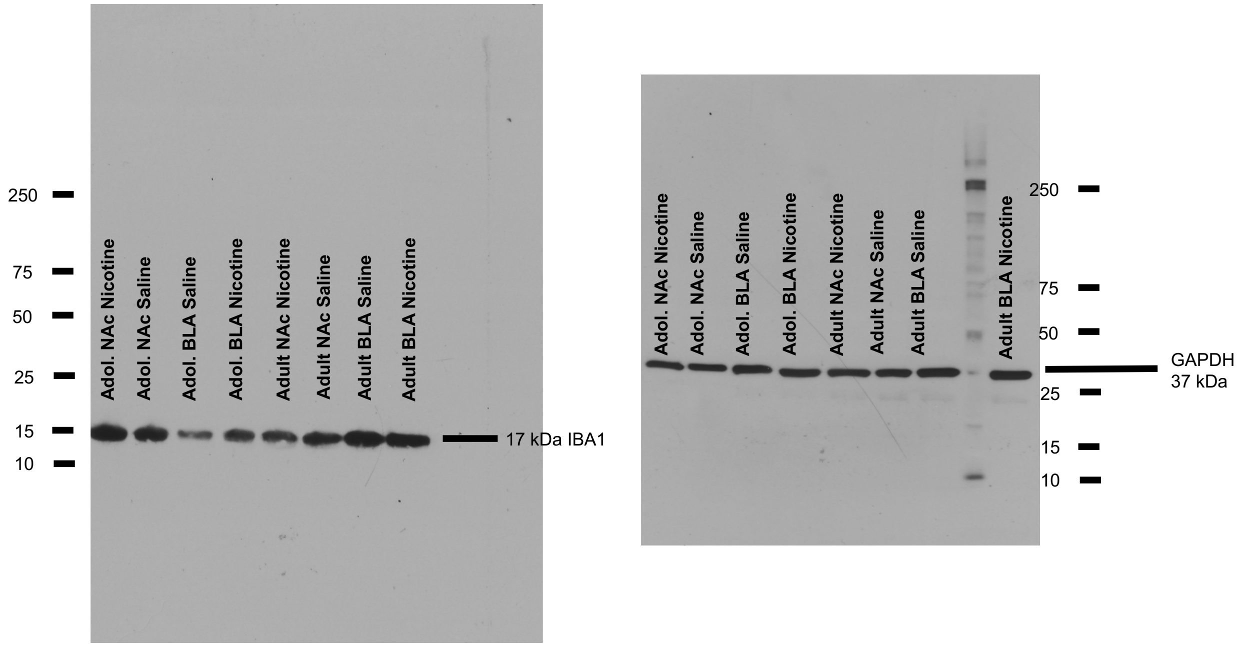

Synaptophysin Western Blot

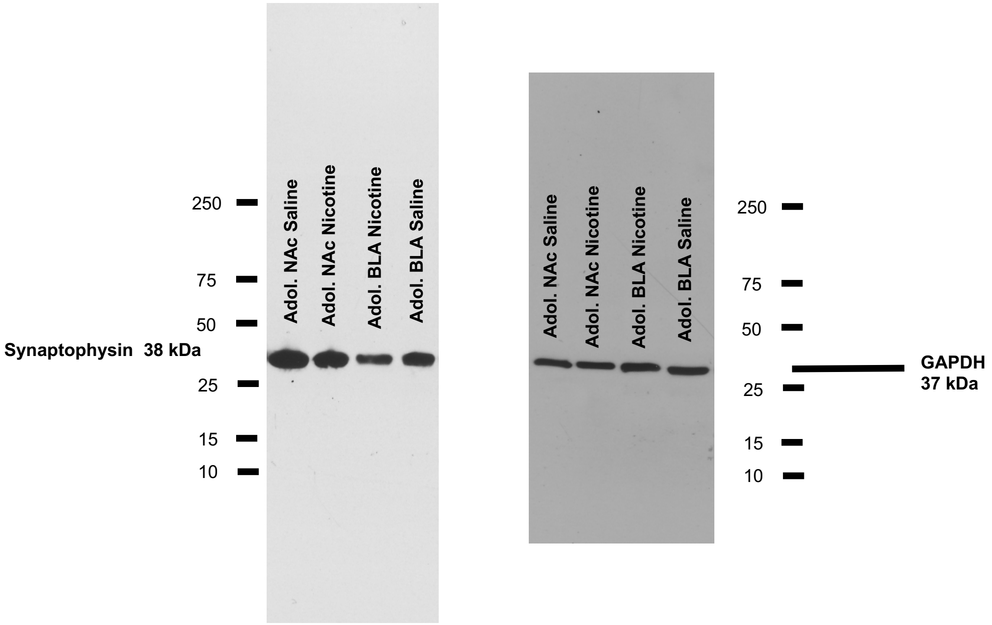

DRD2 Western Blot

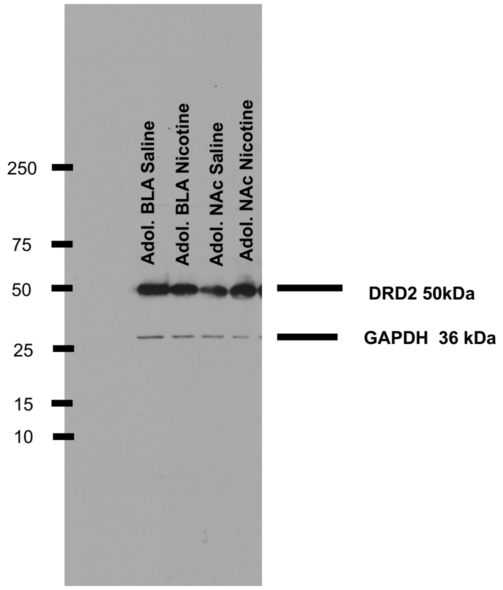

Supplementary Fig. 11: Representative full western blots with markers for IBA1, DRD2 and synaptophysin. Analyzed western data shown in supplementary figure 2.
