# Supplementary material for: Microglial activation increases cocaine self-administration following adolescent nicotine exposure
Source: Nat Commun. 2020 Jan 16;11:306. doi: 10.1038/s41467-019-14173-3 (PMC6965638; doi:10.1038/s41467-019-14173-3)
Supplement: Supplementary file 3 — Reporting Summary [file 41467_2019_14173_MOESM3_ESM.pdf]

nature research

Corresponding author(s): DBPR

Reporting Summary

Nature Research wishes to improve the reproducibility of the work that we publish. This form provides structure for consistency and transparency in reporting. For further information on Nature Research policies, see Authors & Referees and the Editorial Policy Checklist.

Statistical parameters

When statistical analyses are reported, confirm that the following items are present in the relevant location (e.g. figure legend, table legend, main text, or Methods section).

n/a	Confirmed	
☐	☒	The exact sample size (*n*) for each experimental group/condition, given as a discrete number and unit of measurement
☐	☒	An indication of whether measurements were taken from distinct samples or whether the same sample was measured repeatedly
☐	☒	The statistical test(s) used AND whether they are one- or two-sided<br>*Only common tests should be described solely by name; describe more complex techniques in the Methods section.*
☐	☒	A description of all covariates tested
☐	☒	A description of any assumptions or corrections, such as tests of normality and adjustment for multiple comparisons
☐	☒	A full description of the statistics including central tendency (e.g. means) or other basic estimates (e.g. regression coefficient) AND variation (e.g. standard deviation) or associated estimates of uncertainty (e.g. confidence intervals)
☐	☒	For null hypothesis testing, the test statistic (e.g. *F*, *t*, *r*) with confidence intervals, effect sizes, degrees of freedom and *P* value noted<br>*Give P values as exact values whenever suitable.*
☒	☐	For Bayesian analysis, information on the choice of priors and Markov chain Monte Carlo settings
☒	☐	For hierarchical and complex designs, identification of the appropriate level for tests and full reporting of outcomes
☐	☒	Estimates of effect sizes (e.g. Cohen's *d*, Pearson's *r*), indicating how they were calculated
☐	☒	Clearly defined error bars<br>*State explicitly what error bars represent (e.g. SD, SE, CI)*

Our web collection on statistics for biologists may be useful.

Software and code

Policy information about availability of computer code

Data collection	Behavior data: Med associates; Miscellaneous behavioral and molecular data: excel; Nanostring data: nSolver
Data analysis	Behavioral and molecular analysis: Prism 7; Nanostring transcript analysis: nSolver; power analysis: G*Power, cellular morphology:IMARIS software

For manuscripts utilizing custom algorithms or software that are central to the research but not yet described in published literature, software must be made available to editors/reviewers upon request. We strongly encourage code deposition in a community repository (e.g. GitHub). See the Nature Research guidelines for submitting code & software for further information.

Data

Policy information about availability of data

All manuscripts must include a data availability statement. This statement should provide the following information, where applicable:

- Accession codes, unique identifiers, or web links for publicly available datasets
- A list of figures that have associated raw data
- A description of any restrictions on data availability

All data are available from corresponding author upon reasonable request.

Field-specific reporting

Please select the best fit for your research. If you are not sure, read the appropriate sections before making your selection.

☒ Life sciences ☐ Behavioural & social sciences ☐ Ecological, evolutionary & environmental sciences

For a reference copy of the document with all sections, see nature.com/authors/policies/ReportingSummary-flat.pdf

Life sciences study design

All studies must disclose on these points even when the disclosure is negative.

Sample size	No statistical methods were used to pre-determine sample sizes, but our sample sizes are similar to those generally used in the field, including those reported in previous publications.
Data exclusions	Statistical outliers were excluded from the study. For all experiments, values +/-2SD from the group mean were considered outliers and were removed from analyses. Animals that displayed sickness behavior were removed from the study.
Replication	We replicated key findings to ensure reproducibility.<br>-Nicotine increases cocaine responding only in adolescents Fig1); Nicotine increases adolescent microglia cell count, but not adult (Fig.1)<br>-Nicotine promotes a reactive phenotype in adolescent microglia morphology, but a ramified adult NAc microglia morphology (Fig. 2)<br>-Minocycline and PLX3397 treatment blocks increases in cocaine use after adolescent nicotine exposure (Fig. 4)<br>-Raclopride and D2 RNAi blocks nicotine-induced increases in cocaine reinforcement and microglial activation (Fig. 5)<br>- CX3CL1 RNAi blocks nicotine-induced increases in cocaine reinforcement and microglial activation (Fig. 6)<br>-Nicotine decreases synaptophysin levels, and this is blocked by both raclopride, minocycline and PLX (Fig. 7)<br>All experiments that were replicated were successful.
Randomization	All groups were assigned randomly.
Blinding	Image based quantification was performed blindly.

Reporting for specific materials, systems and methods

Materials & experimental systems

n/a	Involved in the study	
☐	☒	Unique biological materials
☐	☒	Antibodies
☒	☐	Eukaryotic cell lines
☒	☐	Palaeontology
☐	☒	Animals and other organisms
☒	☐	Human research participants

Methods

n/a	Involved in the study	
☒	☐	ChIP-seq
☒	☐	Flow cytometry
☒	☐	MRI-based neuroimaging

Unique biological materials

Policy information about availability of materials

Obtaining unique materials	The CSF1R inhibitor PLX3397 was kindly provided by Plexxikon. This compound can also be purchased from chemgood, selleckchem and other third party sellers.

Antibodies

Antibodies used	Primary<br>-rabbit anti IBA1 (1:1000; Wako-Chem; 019-1974)<br>- rabbit anti Synaptophysin, (1:250; Abcam; ab32127)<br>- rabbit DRD2 (1:500; Merk Millipore AB508P)<br>- chicken GFAP (Abcam; ab4674)<br>Secondary<br>-goat-anti rabbit IgG Alexa fluor 488, (1:500, 1:1000; Abcam ab150077)
Validation	Extensive validation data (qPCR, antibody titration, cross-reactivity testing, Western blot analysis, and immunofluorescence) for

this commercial antibody is provided by the vendor. DRD2 antibody has been further confirmed by independent k.o. studies (DRD2: Stojanovic et al., 2017)

Animals and other organisms

Policy information about studies involving animals; ARRIVE guidelines recommended for reporting animal research

Laboratory animals	Sprague Dawley rats were purchased from Jackson Laboratory. Male and Female rats were aged from P15-18 OR P75-P90
Wild animals	NONE
Field-collected samples	NONE
