# Supplementary material for: Microglial activation increases cocaine self-administration following adolescent nicotine exposure
Source: Nat Commun. 2020 Jan 16;11:306. doi: 10.1038/s41467-019-14173-3 (PMC6965638; doi:10.1038/s41467-019-14173-3)
Supplement: Supplementary file 2 — Peer Review File [file 41467_2019_14173_MOESM2_ESM.pdf]

Editorial Note: This manuscript has been previously reviewed at another journal that is not operating a transparent peer review scheme. This document only contains reviewer comments and rebuttal letters for versions considered at *Nature Communications.*

Reviewers' comments:
Reviewer #1 (Remarks to the Author):

The authors have improved the paper with the addition of the new CX3CL1. This important data on its own, but also serves to support the neuronal localization of the D2R. The synaptic staining data (synaptophysin and VGLUT) are interesting but still a bit perplexing as they are not support by the now deleted ephys data on basal transmission. But the authors limit their conclusions and handle these data appropriately. The rest of the finding justify publication.
